# HemoglobinA1c Is a Risk Factor for Changes of Bone Mineral Density: A Mendelian Randomization Study

**DOI:** 10.3389/fendo.2022.942878

**Published:** 2022-07-18

**Authors:** Xiaoxiao Ji, Jianqiao Hong, Zihao Qu, Weinan Yang, Yibo Wang, Jiyan Lin, Congsun Li, Jie Wang, Haochen Mou, Mingmin Shi, Chenhe Zhou, Wei Wang, Changjian Lin, Shigui Yan, Haobo Wu

**Affiliations:** ^1^ Department of Orthopedic Surgery, The Fourth Affiliated Hospital, International Institutes of Medicine, Zhejiang University School of Medicine, Yiwu, China; ^2^ Department of Orthopedic Surgery, the Second Affiliated Hospital, Zhejiang University School of Medicine, Hangzhou, China; ^3^ Orthopedics Research Institute of Zhejiang University, Hangzhou, China; ^4^ Key Laboratory of Motor System Disease Research and Precision Therapy of Zhejiang Province, Hangzhou, China

**Keywords:** bone mineral density, hemoglobinA1c, hemoglobin, mendelian randomization, osteoporosis

## Abstract

**Background:**

As a valuable blood glucose measurement, HemoglobinA1c (HbA1c) is of great clinical value for diabetes. However, in previous observational studies, studies on its effect on bone mineral density (BMD) have different results. This study aimed to use Mendelian randomization (MR) to assess the effect of HbA1c on bone mineral density and fracture risk, and try to further explore whether this association was achieved through glycemic or non-glycemic factors.

**Methods:**

Take HbA1c measurement as exposure, and BMD estimated from quantitative heel ultrasounds (eBMD) and bone fractures as outcomes. Two-Sample MR Analysis was conducted to assess the causal effect of HbA1C on heel BMD and risk fracture. Then, we performed the analysis using two subsets of these variants, one related to glycemic measurement and the other to erythrocyte indices.

**Results:**

Genetically increased HbA1C was associated with the lower heel eBMD [odds ratio (OR) 0.91 (95% CI 0.87, 0.96) per %-unit, P = 3 × 10−4(IVW)]. Higher HbA1C was associated with lower heel eBMD when using only erythrocytic variants [OR 0.87 (0.82, 0.93), P=2× 10−5(IVW)]; However, when using only glycemic variants, this casual association does not hold. In further MR analysis, we test the association of erythrocytic traits with heel eBMD.

**Conclusion:**

Our study revealed the significant causal effect of HbA1c on eBMD, and this causal link might achieve through non-glycemic pathways (erythrocytic indices).

## Introduction

As a result of the advancement and development of society, people’s lifespans have greatly increased, and diseases associated with population aging have become an increasing problem for all humans. As a prevalent senile illness, osteoporosis is defined by decreasing bone strength, which frequently increases the risk of fractures (1, 2). Moreover, the resulting clinical and economic consequences are enormous and intolerable (3). Numerous risk factors for osteoporosis have been identified by previous research, including age, gender, body size, race, family history of fractures, use of certain drugs, smoking, low peak bone mass, low physical activity, and low vitamin D intake (4). In order for patients to have a better prognosis, it is crucial and useful to identify potential risk factors, followed by early clinical evaluation and preventative measures.

As the gold standard for long-term monitoring of glycemic management, HbA1c (hemoglobin A1C) reflects the average amount of glucose in the blood over the past two to three months (5).

Also, it is a reference signal for the risk of some diabetic complications, particularly cardiovascular illnesses (6). However, the effect of HbA1c on bone mineral density and fracture risk has not been established; the diverse or even contradictory outcomes in observational studies are intriguing. High levels of HbA1c have been linked to decreased bone mineral density (7–9) and an increased risk of fracture (10). Other studies, however, have revealed that patients with a higher HbA1c level tend to have a greater Femoral Neck bone mineral density (11).

These contradictory findings demonstrate that observational studies are frequently prone to confounding and bias due to a variety of reasons.

Mendelian randomization analysis can provide strong evidence for the influence of modifiable risk variables on disease or poor health, so overcoming some of the limitations of traditional observational studies. To explore causal correlations between risk factor “exposure” and disease “outcome” (12), this study uses publicly accessible results from large genome-wide association studies (GWAS), utilizing germline genetic variation as an instrumental variable (IV) for exposure.

In this study, HbA1c was chosen as an instrumental variable (IV) in order to examine its causal effect on eBMD and fracture risk. Since HbA1c levels are not only determined by glycemia, but also by erythrocyte-intrinsic nonglycemic determinants of HbA1C (13, 14), we investigated whether this relationship is driven by glycemic or erythrocytic variables.

## Materials and Methods

### Study Design and Data Sources

HbA1c, measured in mmol/mol using HPLC (Bio-Rad Variant II Turbo analysers, Bio-RadLaboratories, USA) and generally reported as a NGSP (National Glycohemoglobin Standardization Program) percent (15). Regarding eBMD and fracture risks, they were determined by bone mineral density estimated from quantitative heel ultrasounds and from bone fractures respectively. The fracture cases were characterized using the 10th version of the International Classification of Diseases codes. Malignant pathological fractures, atypical femoral fractures, periprosthetic fractures and healed fractures were exclude. And the fracture data were questionnaire-based self-reported fractures and from Hospital Episodes Statistics (16).

In this study, we selected HbA1c as an instrumental variable to investigate its causal effect on eBMD and fracture risk. Since HbA1c variants can be classified as “glycemic” or “erythrocytic” (17).

The data source of HbA1c was obtained from MAGIC (the Meta-Analyses of Glucose and Insulin-Related Traits Consortium), a genome-wide association study (GWAS) of 123665 participants of European ancestry (no overlap with the UK Biobank), and 159940 participants of mixed ancestries without diabetes were included in the original GWAS study. With a similar ratio of males to females, the average age of the participants in this GWAS was about 50 years, it also took into account age and gender, as well as study-specific covariates and genetic controls (17).

The data sources of eBMD and fracture were downloaded from the Genetic Factors for Osteoporosis Consortium website (GEFOS, http://www.gefos):. And we choose the largest GWAS of BMD to date that consisted of 426824 white British UK Biobank participants. Regarding to eBMD, with 233185 females and 193639 males, the average age of the white British participants in this GWAS was about 57 years; About fracture, its study data included 53184 positive cases and 373611 controls of white British participants, the average age was about 58.2 years (39-73 years old) (16).

These data sources were showed in **Table 1**.

**Table 1:** Data sets of GWAS used in the MR analysis to estimate the causal effect of HbA1c on eBMD and Fracture.

Exposure or outcome	Studies	Population	Sample size, n	Phenotype	Publication
HbA1c	MAGIC *	Multinational	159,940 participants without diabetes**^†^**	Measured HbA1c by NGSP percent	Wheeler et al. (17)
eBMD	UK Biobank	European	426,824 white British UK Biobank participants	Bone mineral density estimated from quantitative heel ultrasounds	Morris et al. (16)
Fracture	UK Biobank	European	53,184 positive cases and 373,611 controls of white British participants	Include Questionnaire based self-reported fractures and fracture data from Hospital Episodes Statistics	Morris et al. (16)

*MAGIC: the Meta-Analyses of Glucose and Insulin-Related Traits Consortium.

^†^European-only effect estimates for HbA1C were used for this MR analysis (n = 123,665).

### Assumptions

Three core IV assumptions for this Mendelian randomization analysis (relevance, independence and exclusion restriction):

1. The genetic instruments should be associated with the exposure, here HbA1c.
2. The genetic instruments should not be confounded by factors affecting the exposure-outcome relationship.
3. The genetic instruments should only affect the outcome (eBMD) *via* affecting the exposure (HbA1c).

### Genetic Instrumental Variables Selection and Validation

The genetic instrumental variables (IVs) of HbA1c were selected from the genome-wide association study (GWAS) of 123,665 participants of European ancestry (no overlap with the UK Biobank) (17). Here, we process instrumental variables as follows: At first, IVs associated with HbA1c should reach genome-wide significance (P < 5 ×10−8). Secondly, since IVs in strong LD (linkage disequilibrium) may result in skewed findings, it is important to make certain that none of the exposure’s IVs are in LD. The clumping process (R2 < 0.001, window size = 10,000 kb) was performed among the 1000G European reference panel to exclude the IVs with strong LD. Thirdly, these above-selected IVs were extracted from the eBMD and fracture associated GWAS summary statistics. If a particular requested SNP (associated with HbA1c, set as target SNP) is not presented in the outcome GWAS summary statistics, then a SNP (as a proxy) that is in LD with the target SNP will be searched for instead with LD r2 > 0.8. Then the proxy SNP was returned on the outcome GWAS summary data, along with its effect, the effect allele, and the corresponding allele for the target SNP. Fourthly, the harmonization of the effect was performed to make certain that the same SNP effect of exposure and outcome data correspond to same allele. [harmonise_data(exposure_dat, outcome_dat, action = 2)] The detailed screening steps are in **Supplementary Table 1**. After these rigorous selections, these SNPs were used for subsequent analysis.

The selected instrumental SNPs should substantially connect with exposure, we assessed the F statistic to see if there was a weak instrumental variable bias, meaning that the genetic variations chosen as instrumental factors had a weak correlation with exposure. All IVs’ F statistic is greater than 30, which showed in **Supplementary Table 2**, so the possibility of weak instrumental variable bias is small (18).

### Mendelian Randomization Estimation

Using the above data, we ran two-sample MR analysis to generate overall estimates of the effect of HbA1c on eBMD. The IVW method combines Wald estimates for each SNP to provide overall estimates of the effect of HbA1c on eBMD using a meta-analysis approach (i.e, the β coefficient of the SNP for eBMD divides by the β coefficient of the SNP for HbA1c) (19). If the IV2 assumption is not violated (no horizontal pleiotropy), or the horizontal pleiotropy is balanced, IVW linear regression can provide an unbiased causal estimate (20).The MR-PRESSO approach detects and corrects outliers in IVW linear regression, and the MR-PRESSO outlier test requires at least half of the variations to be valid, has balanced pleiotropy, and is based on the Instrument Strength Independent of Direct Effect (InSIDE) condition, which states that instrument-exposure and pleiotropic effects are uncorrelated (21). Based on the premise of InSIDE, the MR-Egger regression conducts a weighted linear regression of the result coefficients on the exposure coefficients (22). Even if all of the genetic variants are invalid IVs, the InSIDE assumption yields a valid test of the null causal hypothesis and a consistent causal effect estimate (22). Nevertheless, MR-Egger estimations are subject to error and are heavily influenced by outlying genetic variants. The Weighted Median approach, which does not require the InSIDE assumption, has been shown to outperform the MR-Egger estimate because of its better causal effect detection power and lesser type I error (23). Therefore, the main results are based on the IVW and WM methods.

### Sensitivity Analysis

The MR-Egger method can determine if genetic variants have pleiotropic effects on outcomes that differ from zero on average (24), so it was used to examine the potential pleiotropic effects of the SNPs we selected. The MR‐PRESSO recognizes the existence of variant effect sizes that are outliers then removing them to correct pleiotropy (21), and it seeks to reduce heterogeneity in the assessment of the causal effect by eliminating SNPs that contribute disproportionately more than expected. In the MR-PRESSO study, the number of distributions was set to 1000. To determine heterogeneity, we employed the IVW and MR-Egger regression. The heterogeneities were measured using the Cochran Q statistic, with a P value of 0.05 indicating significant heterogeneity. Furthermore, we performed a “leave-one-out” sensitivity analysis to identify potentially influential SNPs.

### Procedures of MR Analysis

Firstly, all the above-selected SNPs were performed with MR analysis, If the MR-PRESSO analysis reveals a significant horizontal pleiotropy, the outlier variants will be removed (with a P-value less than the MR-PRESSO outlier test threshold). After this step, if the heterogeneity was still significant, we continued removing other SNPs with P values less than 1 from small to large in the MR-PRESSO outlier test and repeat MR analysis until there was no heterogeneity. Furthermore, in order to identify potentially relevant SNPs, we use the “leave-one-out” sensitivity analysis in which the MR was repeated but each SNP was removed one at a time.

### Software

Based on the TwoSampleMR Guideline and the MR-PRESSO vignette, all statistical analyses were conducted with TwoSampleMR package (v0.5.6) and MRPRESSO package (v1.0) performed in R version 4.0.3 statistical software (using RStudio v1.3.1093).

## Results

### Causal Effect of Increased HbA1c on Lower eBMD

**Figure 1:**
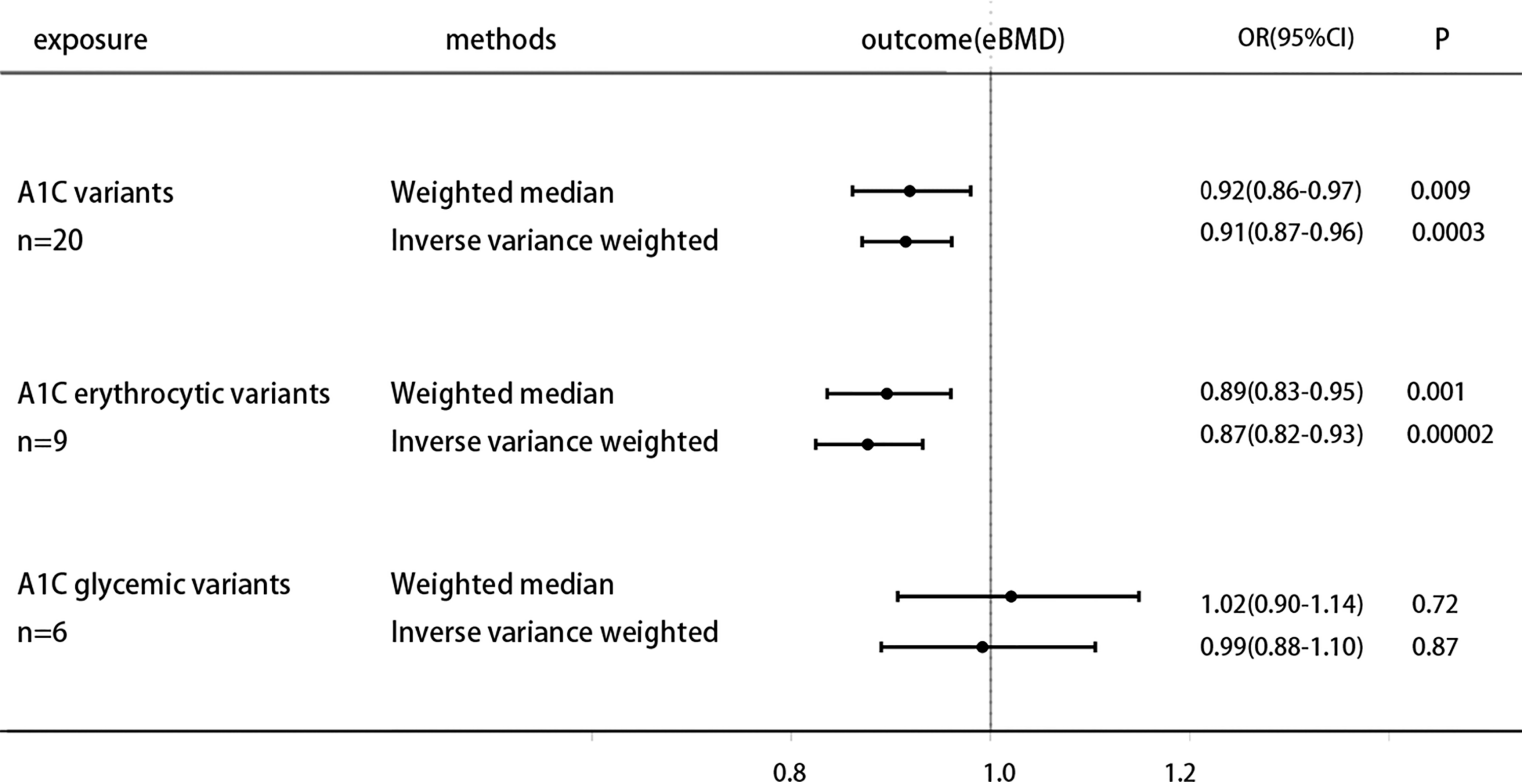
Causal effect on eBMD in UKBB of increased A1C instrumented by all A1C-associated genetic variants, glycemic-only A1C variants, and erythrocytic-only A1C variants. MR analysis were performed by the IVW and WM methods. Effect estimates are OR of eBMD per %-unit increase in A1C.

The specific MR data for all selected SNPs in the exposure set (HbA1c) and outcome set (eBMD) are presented in **Supplementary Table 3**, as well as the exclusion process for subsequent SNPs. The MR estimates from two methods of determining the causal effect of HbA1c on eBMD, which are displayed in **Supplementary Table 4**. Based on the inverse variance weighting (IVW) and Weighted median methods of MR analysis, genetically increased HbA1C is associated with the lower heel eBMD [odds ratio (OR) 0.91 (95% CI 0.87, 0.96), P = 3 × 10−4(IVW); odds ratio (OR) 0.92 (95% CI 0.86, 0.97), P = 9 × 10−3(Weighted median)], the results of the forest plot are shown in Figure 1. The estimated effect sizes of the SNPs on both the HbA1c (exposure) and eBMD(outcome) are presented in scatter plots (**Figure S1A**); The funnel plots, which shows where directional horizontal pleiotropy existed for each result, are displayed in Figure S1B; Plots of the leave-one-out analysis, as shown in **Figure S1C**, demonstrates that no potentially influential SNP that drive the causal effect.

### Causal Effect of HbA1c on eBMD by Erythrocytic Factors

The specific MR data for all selected SNPs in the exposure set (Erythrocytic SNPs) and outcome set (eBMD) are presented in **Supplementary Table 3**, as well as the exclusion process for subsequent SNPs. Higher HbA1C is associated with lower heel eBMD when using only erythrocytic variants [OR 0.87 (0.82, 0.93), P=2× 10−5 (IVW); OR 0.89 (0.83, 0.95), P=1 × 10−3(Weighted median)]. However, when using only glycemic variants, this casual association does not hold [OR 0.99 (0.88, 1.10), P=0.87 (IVW); OR 1.02 (0.90, 1.14), P=0.72 (Weighted median)]. The results of the forest plot are shown in **Figure 1**. The estimated effect sizes of the SNPs on both the erythrocytic variants of HbA1c (exposure) and eBMD (outcome) are presented in scatter plots (**Figure S2A**); The funnel plots, which shows where directional horizontal pleiotropy existed for each result, are displayed in **Figure S2B**; Plots of the leave-one-out analysis, as shown in **Figure S2C**, demonstrates that no potentially influential SNP that drive the causal effect.

### MR Analysis for Association of HbA1c With Fracture

**Figure 2:**
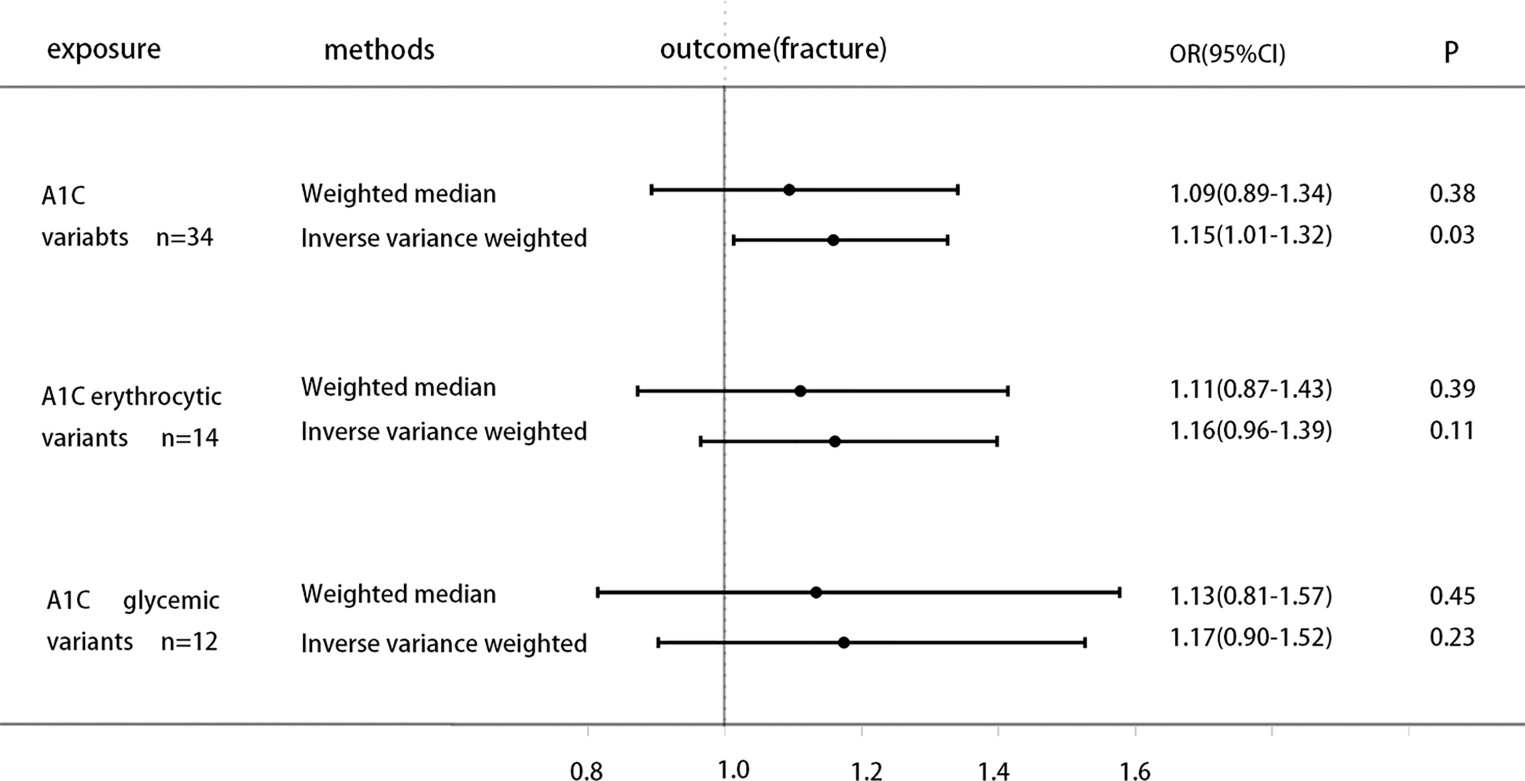
Causal effect on Bone fracture in UKBB of increased A1C instrumented by all A1C-associated genetic variants, glycemic-only A1C variants, and erythrocytic-only A1C variants. MR analysis were performed by the IVW and WM methods. Effect estimates are OR of Bone fracture per %-unit increase in A1C.

The specific MR data for all selected SNPs in the exposure set (Erythrocytic SNPs) and outcome set (Bone Fracture) are presented in **Supplementary Table 3**, as well as the exclusion process for subsequent SNPs. Genetically increased HbA1c is associated with higher Bone fracture [odds ratio (OR) 1.15 (95% CI 1.01, 1.32), P = 0.03(IVW)], while weighted median results [odds ratio (OR) 1.09 (95% CI 0.89, 1.34), P = 0.38] not strictly statistically significant. Moreover, when using the instruments of erythrocytic and glycemic HbA1c variants separately, MR analysis shows that both subtype variants are generally positively associated with fracture risk, but neither reaches statistical significance (**Figure 2**).

### Causal Effect of Hb and Other Erythrocytic Traits on eBMD

**Figure 3:**
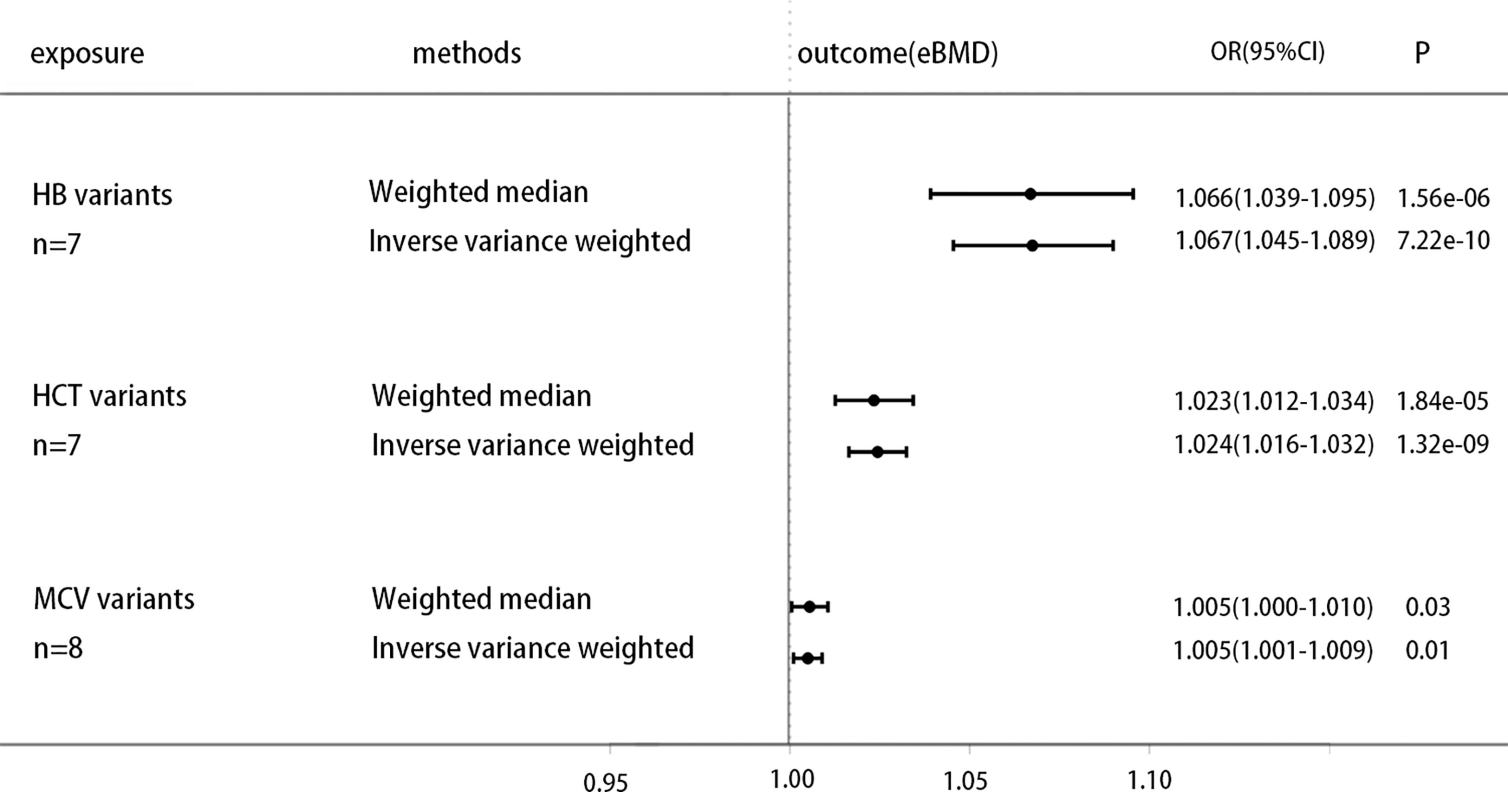
Causal effect on eBMD in UKBB of increased erythrocytic traits, including genetic variants of Hb, HCT and MCV. MR analysis were performed by the IVW and WM methods. Effect estimates are OR of eBMD per %-unit increase in erythrocytic traits.

Previous study finds that genetically decreased Hb is associated with higher A1C (25). To further clarify the causal effect of the non-glycemic fraction of HbA1c on eBMD, exploring the direct causal link of hemoglobin on eBMD may bring us some new inspirations. We select the IVs of Hb and other erythrocytic traits (26) to research their association with eBMD. All analysis procedures are presented in **Supplementary Table 5**. The analysis show that genetically increased hemoglobin and other erythrocytic traits are generally positively associated with increased eBMD, increased hemoglobin is associated with higher heel eBMD [OR 1.066 (1.039, 1.095), P=1.56E-06 (IVW); OR 1.067 (1.045, 1.089), P=7.22E-10(Weighted median)], increased HCT is associated with higher heel eBMD [OR 1.023 (1.012, 1.034), P=1.84E-05 (IVW); OR 1.024 (1.016, 1.032), P=1.32E-09(Weighted median)], increased MCV is associated with higher heel eBMD [OR 1.005 (1.000, 1.010), P=0.03 (IVW); OR 1.005 (1.001, 1.009), P=0.01(Weighted median)], these results of the forest plot are shown in **Figure 3**.

## Discussion

**Figure 4:**
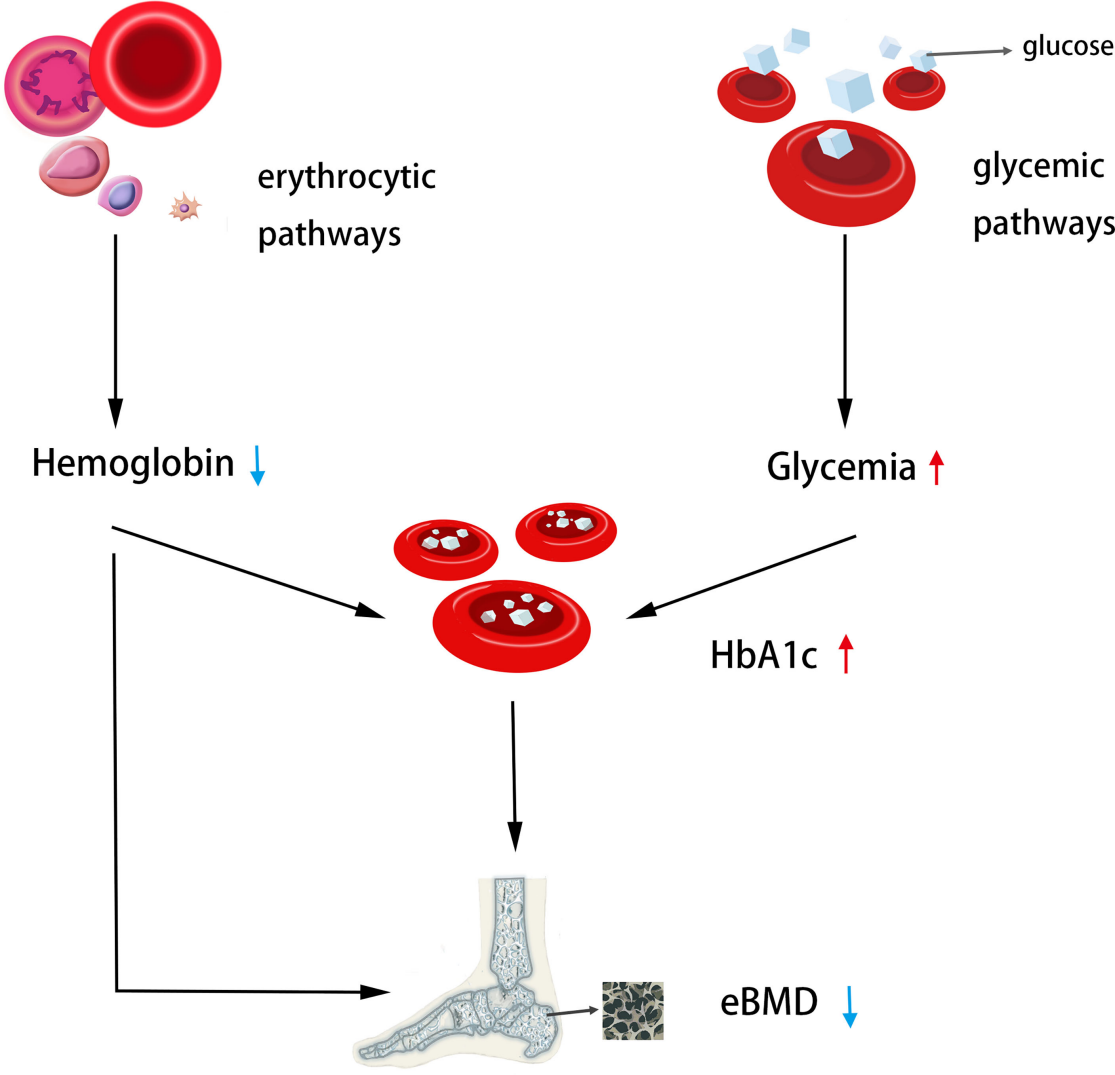
The genetic casual effect of HbA1c on eBMD is represented by an MR diagram of glycemic and erythrocytic factors. Generally, increased HbA1C when instrumented by all HbA1c genetic variants was associated with lower heel eBMD; Increased HbA1c when instrumented by erythrocytic HbA1c variants was associated with lower heel eBMD. Moreover, decreased hemoglobin was associated with lower heel eBMD. The causal association of lower Hb with higher HbA1c has been shown in the literature (25), so the MR analysis was not performed.

Despite the fact that previous research (27, 28) have demonstrated the significance of fracture prevention in diabetics, it is unknown which diabetes-related variables increase or decrease osteoporosis and fracture risk. Hemoglobin A1c (HbA1c) is the gold standard for monitoring long-term glycemic management and reflects the average blood glucose over the previous 2-4 months. In addition, it is easier to determine the HbA1c levels of patients than to perform an oral glucose tolerance test, which is independent of the patient’s prandial status (5). Consequently, the HbA1c level is commonly used as a therapy guideline for the prevention of cardiovascular problems in diabetes patients (6). This relationship between HbA1c and cardiovascular illnesses is further supported by earlier MR investigations (25, 29, 30). Therefore, in this investigation, we chose HbA1c as an instrumental variable to determine if a causal relationship exists between bone mineral density and fracture. In the present study, we provide genetic evidence for HbA1c as a causal predictor of eBMD decline risk *via* exposure (MAGIC) and outcome (UKBB) variables primarily from European populations, and a non-statistically significant positive correlation trend between higher HbA1c and higher fracture risk, which is consistent with the most recent clinical observational findings. At least two recognized subphenotypes are related with genetically high HbA1c, one *via* the diabetic pathway and the other *via* the erythrocyte pathway. To determine if HbA1c as a risk factor impacts eBMD *via* both pathways, we conducted another research. The results demonstrated that this causal association remained when only erythrocyte-related variations were employed, but not when only blood glucose-related variants were used. In a subsequent GWAS analysis, we identified instrumental factors associated with Hb and other erythrocyte characteristics (PAGE STUDY). It was demonstrated through Mendelian experiments that genetically enhanced Hb was positively connected with increased eBMD. It exists also in the HCT and MCV variables. Previous MR investigations have showed that hereditary decreased Hb is associated with elevated HbA1c levels; therefore, the genetic effect of Hb and HbA1c on eBMD was revealed in this work (**Figure 4**).

This discovery has numerous intriguing ramifications:

The results of this investigation can partially explain the contradictory findings of earlier observational studies. In epidemiological research evaluating the effect of HbA1c on bone mineral density, numerous confounding variables, such as gender, age, BMI, etc., must frequently be addressed. After these modifications, some studies have indicated that HbA1c causes a decline in bone mineral density, whereas other studies have found that it causes an increase in bone mineral density or that there is no association between the two. However, none of these studies accounted for Hb levels as a confounding variable, and our findings indicate that HbA1c is a risk factor for lower eBMD, predominantly *via* erythrocytic pathways. When conducting research on HbA1c, it is vital to evaluate the influence of Hb and other erythrocytic features.

Second, HbA1c, which is regarded as the gold standard for predicting risks associated with consequences of diabetes mellitus, has showed incontestable value in microvascular and macrovascular illnesses (31). Higher HbA1c variability in adolescents with T1D is predictive with retinopathy, early nephropathy, and cardiac autonomic neuropathy (32). It indicates that controlling HbA1c levels can prevent proliferative retinopathy and persistent macroalbuminuria for up to 20 years (33). Increased HbA1c is associated with diabetic peripheral neuropathy in individuals with T2D and could be utilized as a valid predictor of diabetic peripheral neuropathy in these patients (34). HbA1c remains the primary glycemic indicator in diabetic individuals with severe chronic renal disease despite its limitations. A suitable HbA1c range can aid in the development of a glycemic control regimen and mitigate the risk of mortality and hypoglycemia (35). Similarly, diabetes-related osteoporosis issues have garnered increasing attention in recent years. Our study provides genetic evidence and support for HbA1c as a possible predictor and control indicator, but further clinical research is required to determine the precise control range and level.

In addition, our study reveals that genetically greater Hb levels are related with higher heel eBMD, as previously documented by studies of a similar nature. In postmenopausal women, low BMD is associated with low Hb levels and the incidence of anemia, according to a cross-sectional study (36). Men’s Hb levels and BMD were found to be positively correlated in a separate cross-sectional study (37). Correction of low Hb levels may have a preventative role in osteoporosis prevention (38). However, there are few findings on the effect of Hb decrease on osteoporosis when the group consists of diabetes people. Alternatively, diabetics are more likely to develop anemia and osteoporosis (39). Therefore, study is required on the relationship between anemia and osteoporosis in diabetics. A cross-sectional retrospective investigation revealed that men and women with the lowest hemoglobin levels had a higher incidence of osteoporosis. And BMD was also associated with both male and female hemoglobin levels (40). They warn diabetics with anemia (men with hemoglobin below 120 g/L and women with hemoglobin below 110 g/L) to be cautious of osteoporosis.

In certain respects, our research results support this hypothesis. This effect occurs mostly through erythrocyte pathways such as Hb decrease, therefore we should pay attention to and prevent osteoporosis complications in patients with higher HbA1c levels, particularly in the presence of certain blood diseases (such as anemia and other diseases that may lead to a decrease in hemoglobin).

However, there are some limitations to this research. Although the causal effect of exposure on outcome was consistent among the MR statistical methods IVW and WM, the results of MR-Egger were less persuasive. Horizontal pleiotropy was detected and corrected by MR-PRESSO; each instrumental variable was determined to be not a weak instrumental variable by F test (all greater than 30, it is generally accepted that greater than 10 is not a weak instrumental variable), and it was reported that the estimated efficiency of MR-Egger is lower than that of WME and IVW (23), so MR-PRESSO was used to correct for horizontal pleiotropy. Second, our findings were restricted to the mineral density of the heel bone. When we use outcome sets of BMD at other body sites (Femoral Neck bone mineral density; Lumbar Spine bone mineral density; Forearm bone mineral density), the relationship between HbA1c and BMD was not statistically significant, so it should be cautious to extrapolate the causal relationship of HbA1c on the heel BMD to whole-body BMD, and we hypothesize that there may be site differences in the effect of HbA1c on BMD, the reasons for which require further investigation. Thirdly, our findings refer to the effects of HbA1c in the non-diabetic range; it is impossible to make conclusions on the genetic impact of HbA1c in diabetics; and we advise care when extrapolating our findings to the extremities of the HbA1c distribution. Therefore, it is more rigorous and scientific to use it as a genetic hypothesis reference in clinical diabetes research. Fourthly, while researching the causal effect of genetically changed Hb levels and other erythrocytic features on eBMD, we cannot extrapolate results to specific blood illnesses or a specific form of anemia, necessitating additional research. This study is limited to the European population; additional research is required to see whether this link exists in other groups.

## Data Availability Statement

The original contributions presented in the study are included in the article/**Supplementary Material**. Further inquiries can be directed to the corresponding authors.

## Author Contributions

XJ: original idea, data acquisition and analysis, and manuscript writing. JH: data acquisition and analysis, and prepared the figures and tables. ZQ: data analysis and manuscript editing. WY, YW, JL, CoL, JW, HM, MS, CZ, WW and ChL made comments and manuscript editing. SY and HW: project development and manuscript editing. All authors contributed to the article and approved the submitted version.

## Funding

This study is supported by research grants from Zhejiang Natural Science Foundation (No. LQ21H060006), Zhejiang Province Medical and Health project (NO.2020391395), the National Natural Science Foundation of China (No.82001461), and the fellowship of China Postdoctoral Science Foundation (No.2020M671758).

## Conflict of Interest

The authors declare that the research was conducted in the absence of any commercial or financial relationships that could be construed as a potential conflict of interest.

## Publisher’s Note

All claims expressed in this article are solely those of the authors and do not necessarily represent those of their affiliated organizations, or those of the publisher, the editors and the reviewers. Any product that may be evaluated in this article, or claim that may be made by its manufacturer, is not guaranteed or endorsed by the publisher.
