# Supplementary material for: HemoglobinA1c Is a Risk Factor for Changes of Bone Mineral Density: A Mendelian Randomization Study
Source: Front Endocrinol (Lausanne). 2022 Jul 18;13:942878. doi: 10.3389/fendo.2022.942878 (PMC9339617; doi:10.3389/fendo.2022.942878)
Supplement: Supplementary file 1 [file DataSheet_1.pdf]

Supplementary Table1

Table S1 Characteristics of the 35 genetic variants considered for use in Mendelian Randomization analysis of the effect of A1C on BMD and bone fracture

Variants							A1C (MAGIC) N=123,665				
RSID	Effect allele	Other allele	Chromosome	Position (CRCh37)	Locus	Classification	EAF	BETA	SE	P	CHOOSE
rs11954649	G	C	5	156988069	SOX30	Unclassified	Data not available	NA	NA	NA	exclude 1
rs1050828	T	C	23	153417411	G6PD	Erythrocytic	Data not available	NA	NA	NA	exclude 1
rs2110073	T	C	12	6946143	PHB2	Unclassified	0.1097	0.015	0.0028	4.44E-08	exclude 2
rs6980507	A	G	8	42502241	SLC20A2	Erythrocytic	0.4014	0.010	0.0018	3.58E-08	exclude 2
rs9604573	T	C	13	113571085	GAS6	Unclassified	0.2738	0.010	0.0018	9.60E-09	exclude 2
rs2408955	T	G	12	46785398	SENP1	Erythrocytic	0.5039	0.012	0.0016	1.42E-15	exclude 2
rs11154792	T	C	6	135473333	MYB	Erythrocytic	0.7415	0.021	0.0024	7.45E-18	exclude 2
rs1800562	G	A	6	26201120	HFE	Erythrocytic	0.9279	0.040	0.0036	4.67E-28	exclude 2
rs3824065	C	T	7	44213783	GCK	Glycemic	0.5557	0.019	0.0016	4.22E-35	exclude 2
rs10823343	A	G	10	70761019	HK1	Unclassified	0.7223	0.033	0.0021	1.68E-55	exclude 2
rs13387347	T	C	2	169463092	G6PC2	Glycemic	0.457	0.002	0.0019	3.08E-01	exclude 3
rs12132919	A	C	1	154584765	TMEM79	Erythrocytic	0.3081	0.005	0.002	1.69E-02	exclude 3
rs2237896	G	A	11	2815016	KCNQ1	Glycemic	0.9159	0.012	0.0038	2.46E-03	exclude 3
rs17256082	C	T	2	175000610	SCRN3	Unclassified	0.364	0.005	0.0017	1.12E-03	exclude 3
rs2073285	C	T	17	73628956	TMC6	Unclassified	0.7517	0.013	0.0033	1.27E-04	exclude 3
rs576674	G	A	13	32452302	KL	Glycemic	0.1883	0.010	0.0023	1.39E-05	exclude 3
rs11086054	A	T	19	17107737	MYO9B	Unclassified	0.6753	0.010	0.0022	8.16E-06	exclude 3
rs4894799	A	G	3	173278234	FNDC3B	Unclassified	0.6214	0.009	0.0019	1.80E-06	exclude 3
rs174577	C	A	11	61361390	FADS2	Glycemic	0.6485	0.008	0.0017	5.45E-07	exclude 3
rs11224302	C	T	11	99961814	CNTN5	Erythrocytic	0.869	0.015	0.0029	4.76E-07	exclude 3
rs11619319	G	A	13	27385599	PDX1	Glycemic	0.2327	0.009	0.0019	4.58E-07	exclude 3
rs2191349	T	G	7	15030834	DGKB	Glycemic	0.532	0.009	0.0017	2.09E-07	exclude 3
rs1467311	G	A	9	109576753	KLF4	Unclassified	0.3508	0.009	0.0017	2.09E-07	exclude 3
rs2375278	A	G	1	25401625	SYF2	Unclassified	0.1888	0.011	0.0022	2.03E-07	exclude 3
rs4783565	A	G	16	67307691	CDH3	Erythrocytic	0.2941	0.009	0.0018	1.73E-07	exclude 3
rs592423	A	C	6	139882386	CITED2	Erythrocytic	0.4566	0.009	0.0017	3.96E-08	adopt
rs1558902	A	T	16	52361075	FTO	Unclassified	0.4128	0.010	0.0019	3.27E-08	adopt
rs12621844	T	C	2	48268239	FOXN2	Unclassified	0.5999	0.010	0.0018	1.87E-08	adopt
rs10774625	G	A	12	110394602	ATXN2	Erythrocytic	0.5056	0.009	0.0016	1.46E-08	adopt
rs579459	C	T	9	135143989	ABO	Glycemic	0.2389	0.011	0.0019	9.42E-09	adopt
rs11603334	G	A	11	72110633	ARAP1	Glycemic	0.815	0.012	0.0021	6.85E-09	adopt
rs267738	T	G	1	149207249	CERS2	Unclassified	0.7701	0.011	0.0019	2.59E-09	adopt
rs9818758	A	G	3	49357929	USP4	Unclassified	0.2028	0.012	0.002	7.74E-10	adopt
rs11964178	A	G	6	109668728	C6orf183	Erythrocytic	0.5666	0.010	0.0016	6.38E-10	adopt
rs7616006	A	G	3	12242648	SYN2	Erythrocytic	0.5744	0.010	0.0017	5.07E-10	adopt
rs3782123	C	A	11	195198	BET1L	Unclassified	0.3205	0.013	0.002	1.51E-10	adopt
rs17747324	C	T	10	114742493	TCF7L2	Glycemic	0.2489	0.015	0.0023	6.12E-11	adopt
rs9914988	A	G	17	24207230	ERAL1	Erythrocytic	0.7877	0.013	0.002	2.77E-11	adopt
rs8192675	T	C	3	172207577	SLC2A2	Glycemic	0.6906	0.011	0.0017	1.38E-11	adopt
rs2383208	A	G	9	22122076	MTAP	Glycemic	0.7992	0.014	0.0021	7.04E-12	adopt
rs17533903	A	G	19	17117523	MYO9B	Erythrocytic	0.2428	0.015	0.0022	5.27E-12	adopt
rs7756992	G	A	6	20787688	CDKAL1	Glycemic	0.2862	0.012	0.0018	2.80E-12	adopt
rs282587	G	A	13	112399663	ATP11A	Unclassified	0.1513	0.019	0.0027	1.70E-12	adopt
rs11708067	A	G	3	124548468	ADCY5	Glycemic	0.7542	0.013	0.0019	1.42E-12	adopt
rs7040409	C	G	9	90693056	C9orf47	Erythrocytic	0.8953	0.028	0.0037	2.56E-14	adopt
rs11248914	T	C	16	233563	ITFG3	Erythrocytic	0.647	0.014	0.0019	2.56E-14	adopt
rs13134327	A	G	4	144879245	FREM3	Glycemic	0.3335	0.013	0.0017	2.64E-15	adopt
rs17509001	C	T	2	23874735	ATAD2B	Unclassified	0.1576	0.018	0.0023	1.94E-15	adopt
rs6474359	T	C	8	41668351	ANK1	Unclassified	0.953	0.044	0.0053	1.50E-16	adopt
rs11558471	A	G	8	118254914	SLC30A8	Glycemic	0.6745	0.015	0.0017	1.38E-19	adopt
rs4820268	G	A	22	35799537	TMPRSS6	Erythrocytic	0.4606	0.016	0.0017	1.40E-22	adopt
rs10830963	G	C	11	92348358	MTNR1B	Glycemic	0.2938	0.020	0.002	2.23E-23	adopt
rs198846	G	A	6	26215442	HFE	Erythrocytic	0.8293	0.022	0.0022	1.18E-23	adopt
rs857691	T	C	1	156893002	SPTA1	Erythrocytic	0.2715	0.019	0.0019	3.97E-25	adopt
rs4737009	A	G	8	41749562	ANK1	Erythrocytic	0.2531	0.021	0.002	4.48E-27	adopt
rs837763	T	C	16	87381230	CDT1	Erythrocytic	0.5548	0.017	0.0016	1.68E-28	adopt
rs4607517	A	G	7	44202193	GCK	Glycemic	0.2017	0.031	0.0024	8.76E-38	adopt
rs560887	C	T	2	169471394	G6PC2	Glycemic	0.6843	0.028	0.0018	1.48E-58	adopt
rs1046896	T	C	17	78278822	FN3KRP	Unclassified	0.3162	0.028	0.0017	4.46E-64	adopt
rs4745982	T	G	10	70759849	HK1	Erythrocytic	0.8726	0.095	0.0056	2.87E-65	adopt

exclude 1: Data not available for this SNP in this ancestry

exclude 2 LD(clump_r2=0.001,clump_kb=10000)

exclude 3 P>5e-8

Classification: Classification of A1C variants based on probable biology in Wheeler et al. 2017. A1C variants were classified as 'glycemic' if they were associated with FG or 2-hour glucose (P < 0.0001) from GWAS results(8-10) or had > 25% attenuation of A1C effect size in association models conditioned on FG or 2-hour glucose, suggesting that their association with A1C was likely driven through an association with glycemia. A1C variants were classified as 'erythrocytic' if they were associated with Hb, mean corpuscular volume (MCV), mean corpuscular hemoglobin (MCH), mean corpuscular hemoglobin concentration (MCHC), red blood cell count (RBC), or packed cell volume (PCV) at P < 0.0001 from GWAS results(11), or had > 25% attenuation of effect size in Hb-, MCV-, or MCH-conditioned models, suggesting that their association with A1C was likely driven through an association with these erythrocytic traits.

Supplementary Table2

SNP	R2	F statistic
rs267738	0.010192235	36.37231115
rs857691	0.02982123	108.5740631
rs17509001	0.018464235	66.44732664
rs12621844	0.009408639	33.54938551
rs560887	0.069235518	262.7492328
rs7616006	0.010523384	37.56662585
rs9818758	0.010944525	39.08666273
rs11708067	0.014181321	50.81266155
rs8192675	0.012715404	45.49253246
rs13134327	0.017664026	63.51582694
rs7756992	0.013463865	48.20688403
rs198846	0.02982123	108.5740631
rs1196417[illegible]	0.011875778	42.45245869
rs592423	0.008533162	30.40073768
rs4607517	0.048770279	181.1015373
rs6474359	0.020739171	74.8075295
rs4737009	0.032763337	119.6486176
rs11558471	0.023384856	84.57919519
rs2383208	0.013463865	48.20688403
rs7040409	0.017307958	62.21293818
rs579459	0.010192235	36.37231115
rs4745982	0.081273818	312.4761537
rs17747324	0.012895138	46.14397683
rs3782123	0.012805279	45.81825465
rs11603334	0.009951261	35.50371865
rs1083096[illegible]	0.02982123	108.5740631
rs1077462[illegible]	0.009619729	34.30940395
rs282587	0.014987212	53.74416125
rs11248914	0.016416659	58.95571629
rs1558902	0.008442507	30.07501549
rs837763	0.033539145	122.5801173
rs9914988	0.012805279	45.81825465
rs1046896	0.076972929	294.5614333
rs1753390[illegible]	0.014091696	50.48693936
rs4820268	0.026511739	96.19661994

Supplementary Table3

Variants							A1C (MAGIC) N=123,665				BMD(UKBB) N=426824								BONE FRACTURE (UKBB) N=426795							
RSID	EA	OA	Chr	BP(CRCh3	Locus	Classification	EAF	BETA	SE	P	RSID	EA	OA	EAF	BETA	SE	P	CHOOSE	RSID	EA	OA	EAF	BETA	SE	P	CHOOSE
rs1558902	A	T	16	52361075	FTO	**Unclassified**	0.4128	0.010	0.0019	3.27E-08	rs1558902	T	A	0.59806	-0.019	0.00189	1.20E-19	exclude1	rs1558902	T	A	0.598054	0.014814	0.00667	0.029	exclude4
rs579459	C	T	9	1.35E+08	ABO	**Glycemic**	0.2389	0.011	0.0019	9.42E-09	rs579459	T	C	0.793151	0.0244	0.00229	1.10E-18	exclude1	rs579459	T	C	0.793143	-0.012265	0.008084	0.15	
rs7616006	A	G	3	12242648	SYN2	**Erythrocytic**	0.5744	0.010	0.0017	5.07E-10	rs7616006	A	G	0.582027	-0.014	0.00187	1.30E-13	exclude1	rs7616006	A	G	0.582047	0.012713	0.006629	0.05	
rs17747324	C	T	10	1.15E+08	TCF7L2	**Glycemic**	0.2489	0.015	0.0023	6.12E-11	rs17747324	T	C	0.772937	-0.013	0.00221	3.70E-09	exclude1	rs17747324	T	C	0.772912	-0.009335	0.007832	0.25	
rs11708067	A	G	3	1.25E+08	ADCY5	**Glycemic**	0.7542	0.013	0.0019	1.42E-12	rs11708067	A	G	0.755071	0.013	0.00215	2.50E-07	exclude2	rs11708067	A	G	0.755096	-0.00877	0.007605	0.24	
rs267738	T	G	1	1.49E+08	CERS2	**Unclassified**	0.7701	0.011	0.0019	2.59E-09	rs267738	T	G	0.778812	0.0126	0.00221	6.10E-07	exclude2	rs267738	T	G	0.7788	0.005848	0.007884	0.44	
rs11964178	A	G	6	1.1E+08	C6orf183	**Erythrocytic**	0.5666	0.010	0.0016	6.38E-10	rs11964178	A	G	0.56605	0.0092	0.00185	2.30E-05	exclude2	rs11964178	A	G	0.566061	-0.001963	0.006608	0.71	
rs837763	T	C	16	87381230	CDT1	**Erythrocytic**	0.5548	0.017	0.0016	1.68E-28	rs837763	C	T	0.44547	0.0082	0.00186	2.50E-05	exclude2	rs837763	C	T	0.445476	-0.003445	0.006576	0.65	
rs9818758	A	G	3	49357929	USP4	**Unclassified**	0.2028	0.012	0.002	7.74E-10	rs9818758	G	A	0.830066	0.0104	0.0025	0.00014	exclude2	rs9818758	G	A	0.830072	-0.00646	0.008839	0.48	
rs10830963	G	C	11	92348358	MTNR1B	**Glycemic**	0.2938	0.020	0.002	2.23E-23	rs10830963	C	G	0.724685	-0.009	0.00206	0.00027	exclude2	rs10830963	C	G	0.724662	-0.001687	0.007311	0.84	
rs10774625	G	A	12	1.1E+08	ATXN2	**Erythrocytic**	0.5056	0.009	0.0016	1.46E-08	rs10774625	A	G	0.495833	0.0065	0.00264	0.00089	exclude4	rs10774625	A	G	0.495794	0.002833	0.009358	0.74	
rs4820268	G	A	22	35799537	TMPRSS	**Erythrocytic**	0.4606	0.016	0.0017	1.40E-22	rs4820268	G	A	0.463171	-0.006	0.00186	0.001	exclude3	rs4820268	G	A	0.46316	0.009112	0.006552	0.15	
rs12621844	T	C	2	48268239	FOXN2	**Unclassified**	0.5999	0.010	0.0018	1.87E-08	rs12621844	C	T	0.37815	-0.005	0.00188	0.0015	exclude3	rs12621844	C	T	0.378125	0.003089	0.006735	0.62	
rs198846	G	A	6	26215442	HFE	**Erythrocytic**	0.8293	0.022	0.0022	1.18E-23	rs198846	A	G	0.156502	0.0068	0.00252	0.0039		rs198846	A	G	0.156522	-0.012983	0.009012	0.13	
rs11603334	G	A	11	72110633	ARAP1	**Glycemic**	0.815	0.012	0.0021	6.85E-09	rs11603334	G	A	0.844876	0.0083	0.00254	0.014	exclude3	rs11603334	G	A	0.844866	0.010021	0.009022	0.31	
rs857691	T	C	1	1.57E+08	SPTA1	**Erythrocytic**	0.2715	0.019	0.0019	3.97E-25	rs857691	C	T	0.749675	0.0049	0.00213	0.018		rs857691	C	T	0.749663	0.00604	0.007591	0.45	
rs7040409	C	G	9	90693056	C9orf47	**Erythrocytic**	0.8953	0.028	0.0037	2.56E-14	rs7040409	C	G	0.942164	-0.008	0.00401	0.028		rs7040409	C	G	0.942169	0.017215	0.014157	0.22	
rs2383208	A	G	9	22122076	MTAP	**Glycemic**	0.7992	0.014	0.0021	7.04E-12	rs2383208	A	G	0.821805	-0.004	0.00242	0.048		rs2383208	A	G	0.821828	0.008223	0.008547	0.39	
rs4745982	T	G	10	70759849	HK1	**Erythrocytic**	0.8726	0.095	0.0056	2.87E-65	rs4745982	T	G	0.920619	-0.008	0.00352	0.057		rs4745982	T	G	0.920614	0.008501	0.012491	0.53	
rs560887	C	T	2	1.69E+08	G6PC2	**Glycemic**	0.6843	0.028	0.0018	1.48E-58	rs560887	T	C	0.298468	-0.003	0.002	0.11		rs560887	T	C	0.298479	-0.00655	0.007133	0.33	
rs8192675	T	C	3	1.72E+08	SLC2A2	**Glycemic**	0.6906	0.011	0.0017	1.38E-11	rs8192675	T	C	0.712668	-0.003	0.00204	0.11		rs8192675	T	C	0.712662	-0.000262	0.007213	0.92	
rs9914988	A	G	17	24207230	ERAL1	**Erythrocytic**	0.7877	0.013	0.002	2.77E-11	rs9914988	G	A	0.204386	0.002	0.00229	0.12		rs9914988	G	A	0.20441	-0.00303	0.008098	0.68	
rs11558471	A	G	8	1.18E+08	SLC30A8	**Glycemic**	0.6745	0.015	0.0017	1.38E-19	rs11558471	A	G	0.681652	0.0041	0.00199	0.17	exclude2	rs11558471	A	G	0.681642	-0.005109	0.007023	0.47	
rs4737009	A	G	8	41749562	ANK1	**Erythrocytic**	0.2531	0.021	0.002	4.48E-27	rs4737009	G	A	0.763965	0.003	0.00218	0.25		rs4737009	G	A	0.763989	-0.00321	0.007692	0.7	
rs11248914	T	C	16	233563	ITFG3	**Erythrocytic**	0.647	0.014	0.0019	2.56E-14	rs11248914	T	C	0.664735	-0.003	0.00197	0.32		rs11248914	T	C	0.664732	-0.004673	0.006958	0.45	
rs592423	A	C	6	1.4E+08	CITED2	**Erythrocytic**	0.4566	0.009	0.0017	3.96E-08	rs592423	A	C	0.446211	-0.003	0.00184	0.38		rs592423	A	C	0.446231	0.000296	0.00657	0.98	
rs282587	G	A	13	1.12E+08	ATP11A	**Unclassified**	0.1513	0.019	0.0027	1.70E-12	rs282587	G	A	0.12225	0.0025	0.00283	0.4		rs282587	G	A	0.122268	0.008799	0.009972	0.34	
rs17509001	C	T	2	23874735	ATAD2B	**Unclassified**	0.1576	0.018	0.0023	1.94E-15	rs17509001	T	C	0.854147	0.0032	0.00259	0.42		rs17509001	T	C	0.854136	-0.00115	0.009265	0.94	
rs1046896	T	C	17	78278822	FN3KRP	**Unclassified**	0.3162	0.028	0.0017	4.46E-64	rs1046896	C	T	0.687637	0.0023	0.00199	0.44		rs1046896	C	T	0.687635	-0.000678	0.007059	0.87	
rs7756992	G	A	6	20787688	CDKAL1	**Glycemic**	0.2862	0.012	0.0018	2.80E-12	rs7756992	A	G	0.734967	0.0019	0.00207	0.52		rs7756992	A	G	0.734962	-0.013802	0.007404	0.059	
rs6474359	T	C	8	41668351	ANK1	**Unclassified**	0.953	0.044	0.0053	1.50E-16	rs6474359	T	C	0.962153	-0.004	0.00485	0.54		rs6474359	T	C	0.962159	-0.012113	0.017141	0.48	
rs3782123	C	A	11	195198	BET1L	**Unclassified**	0.3205	0.013	0.002	1.51E-10	rs3782123	C	A	0.266978	-2E-04	0.00209	0.74		rs3782123	C	A	0.266963	0.012017	0.00742	0.097	
rs13134327	A	G	4	1.45E+08	FREM3	**Glycemic**	0.3335	0.013	0.0017	2.64E-15	rs13134327	G	A	0.682476	-0.001	0.00198	0.79		rs13134327	G	A	0.682471	0.008914	0.007017	0.16	
rs4607517	A	G	7	44202193	GCK	**Glycemic**	0.2017	0.031	0.0024	8.76E-38	rs4607517	G	A	0.822341	0.0007	0.00238	0.89		rs4607517	G	A	0.822357	-0.002789	0.00855	0.78	
rs17533903	A	G	19	17117523	MYO9B	**Erythrocytic**	0.2428	0.015	0.0022	5.27E-12	rs17533903	G	A	0.794932	-0.001	0.00231	0.92		rs17533903	G	A	0.794944	-0.000737	0.008125	0.95	

exclude1: P-value in the outcome set is less than 5E-8,the SNP is considered to be directly related to the outcome.

exclude2 Use MR-PRESSO to remove the outlier variants(with a P-value less than the MR-PRESSO outlier test threshold).

exclude3 Continued removing other SNPs with P-values less than 1 from small to large in the MR-PRESSO outlier test and repeat MR analysis until there was no heterogeneity.

exclude4 The funnel plots show significant deviations.

MAGIC: Meta-Analysis of Glycemic and Insulin-related traits Consortium;

Supplementary Table4

MR estimates of HbA1c on eBMD

Method	SNP(N)	BETA	SE	P-value	OR(95%CI)
Weighted median	20	-0.084931066	0.032946535	0.009941852	0.92(0.86-0.97)
Inverse variance weighted	20	-0.089280541	0.025104057	0.000375945	0.91(0.87-0.96)

MR estimates of erythrocytic varients of HbA1c on eBMD

Method	SNP(N)	BETA	SE	P-value	OR(95%CI)
Weighted median	9	-0.110205994	0.03535786	0.001827797	0.89(0.83-0.95)
Inverse variance weighted	9	-0.131960147	0.031186656	2.32E-05	0.87(0.82-0.93)

MR estimates of glycemic varients of HbA1c on eBMD

Method	SNP(N)	BETA	SE	P-value	OR(95%CI)
Weighted median	6	0.020172944	0.05724559	0.724543563	1.02(0.90-1.14)
Inverse variance weighted	6	-0.008457056	0.055166527	0.878161309	0.99(0.88-1.10)

MR estimates of HbA1c on Bone Fracture

Method	SNP(N)	BETA	SE	P-value	OR(95%CI)
Weighted median	34	0.089603403	0.103771895	0.387881837	1.09(0.89-1.34)
Inverse variance weighted	34	0.14709543	0.068655897	0.03215307	1.16(1.01-1.32)

MR estimates of erythrocytic varients of HbA1c on Bone Fracture

Method	SNP(N)	BETA	SE	P-value	OR(95%CI)
Weighted median	14	0.104541835	0.123230346	0.39624592	1.11(0.87-1.41)
Inverse variance weighted	14	0.149147628	0.094711132	0.115311058	1.16(0.96-1.40)

MR estimates of glycemic varients of HbA1c on Bone Fracture

Method	SNP(N)	BETA	SE	P-value	OR(95%CI)
Weighted median	12	0.124775313	0.168743918	0.459642341	1.13(0.81-1.53)
Inverse variance weighted	12	0.160093538	0.134141557	0.232686478	1.17(0.90-1.52)

Supplementary Table5

Variants				RBC traits（PAGE study）							BMD(UKBB)							
RSID	CHR:BP	EA	OA	EAF	BETA	SE	P	TYPE	effN	N	RSID	EA	OA	EAF	BETA	SE	P	Choose
rs855791	22:37462936	G	A	0.5667	0.0993	0.0082	8.49E-34	HGB	13,716	29,509	rs855791	A	G	0.438429	-0.006	0.00188032	0.00056	
rs1800562	6:26093141	G	A	0.9385	-0.1697	0.0166	1.198E-24	HGB	3,360	29,509	rs1800562	G	A	0.92112	0.01162	0.00339748	0.0011	exclude3
rs2032451	6:26092170	G	T	0.8476	-0.1002	0.011	1.168E-19	HGB	7,609	29,509	rs2032451	G	T	0.847802	-0.007	0.00254662	0.0035	
rs17476364	10:71094504	C	T	0.0946	0.126	0.0142	8.687E-19	HGB	4,433	29,509	rs17476364	T	C	0.890487	-0.0082	0.00295339	0.062	
rs597808	12:111973358	G	A	0.5055	-0.0621	0.0081	2.094E-14	HGB	14,093	29,509	rs597808	A	G	0.483353	0.00724	0.00265788	0.00043	
rs11066283	12:112840766	T	A	0.5518	0.0598	0.0082	3.543E-13	HGB	13,814	29,509	rs11066283							exclude1
rs61523591	2:46372781	C	T	0.1463	-0.0781	0.0113	3.985E-12	HGB	7,333	29,509	rs61523591	T	C	0.861789	0.00284	0.00264817	0.13	
rs10253736	7:151415256	C	T	0.7102	0.0562	0.0092	9.97E-10	HGB	10,736	29,509	rs10253736	C	T	0.716396	0.00371	0.00202255	0.067	
rs2519093	9:136141870	C	T	0.803	0.0611	0.0102	1.879E-09	HGB	8,811	29,509	rs2519093	C	T	0.816049	0.02883	0.00239495	6.8E-24	exclude2
rs35786788	6:135419042	G	A	0.739	0.0499	0.0091	4.295E-08	HGB	11,168	29,509	rs35786788	T	C	0.740897	-0.0035	0.00209601	0.12	
rs855791	22:37462936	G	A	0.5667	0.2288	0.0243	4.685E-21	HCT	13,718	29,514	rs855791	A	G	0.438429	-0.006	0.00188032	0.00056	
rs17476364	10:71094504	C	T	0.0946	0.361	0.0422	1.095E-17	HCT	4,433	29,514	rs17476364	T	C	0.890487	-0.0082	0.00295339	0.062	
rs1800562	6:26093141	G	A	0.9385	-0.3819	0.0491	7.404E-15	HCT	3,361	29,514	rs1800562	G	A	0.92112	0.01162	0.00339748	0.0011	
rs35786788	6:135419042	G	A	0.739	0.2012	0.027	9.444E-14	HCT	11,170	29,514	rs35786788	T	C	0.740897	-0.0035	0.00209601	0.12	
rs597808	12:111973358	G	A	0.5055	-0.1679	0.0241	3.127E-12	HCT	14,096	29,514	rs597808	A	G	0.483353	0.00724	0.00265788	0.00043	
rs61523591	2:46372781	C	T	0.1463	-0.2237	0.0334	2.069E-11	HCT	7,339	29,514	rs61523591	T	C	0.861789	0.00284	0.00264817	0.13	
rs11066283	12:112840766	T	A	0.5518	0.1592	0.0244	6.672E-11	HCT	13,815	29,514	rs11066283							exclude1
rs2032451	6:26092170	G	T	0.8476	-0.2134	0.0328	7.363E-11	HCT	7,610	29,514	rs2032451	G	T	0.847802	-0.007	0.00254662	0.0035	
rs10253736	7:151415256	C	T	0.7102	0.1593	0.0272	4.692E-09	HCT	10,738	29,514	rs10253736	C	T	0.716396	0.00371	0.00202255	0.067	
rs2519093	9:136141870	C	T	0.803	0.172	0.030	1.1E-08	HCT	8,826	29,514	rs2519093	C	T	0.816049	0.02883	0.00239495	6.8E-24	exclude2
rs35786788	6:135419042	G	A	0.739	-0.8698	0.058	7.789E-51	MCV	5,568	14,710	rs35786788	T	C	0.740897	-0.0035	0.00209601	0.12	exclude4
rs855791	22:37462936	G	A	0.5667	0.5782	0.0524	2.544E-28	MCV	6,880	14,710	rs855791	A	G	0.438429	-0.006	0.00188032	0.00056	exclude3
rs1800562	6:26093141	G	A	0.9385	-0.9763	0.1049	1.342E-20	MCV	1,685	14,710	rs1800562	G	A	0.92112	0.01162	0.00339748	0.0011	exclude3
rs2032451	6:26092170	G	T	0.8476	-0.6301	0.0706	4.24E-19	MCV	3,788	14,710	rs2032451	G	T	0.847802	-0.007	0.00254662	0.0035	exclude3
rs1410492	6:41907855	G	C	0.75	0.438	0.0589	9.86E-14	MCV	5,367	14,710	rs1410492	G	C	0.738227	-0.0009	0.00208278	0.44	
rs11964516	6:41860252	C	T	0.1774	0.4834	0.0678	1.001E-12	MCV	4,099	14,710	rs11964516	T	C	0.818167	0.00048	0.00236956	1	
rs140523	22:50962782	G	C	0.6126	0.3845	0.0594	9.376E-11	MCV	3,437	14,710	rs140523	C	G	0.390271	-0.0009	0.00190513	0.81	
rs218265	4:55408999	C	T	0.158	0.4577	0.0728	3.149E-10	MCV	3,507	14,710	rs218265	T	C	0.845215	-0.0049	0.0025781	0.28	
rs7385804	7:100235970	C	A	0.3708	-0.3308	0.0545	1.292E-09	MCV	6,382	14,710	rs7385804	C	A	0.376323	0.00396	0.00187994	0.0054	exclude4
rs13203155	6:109614844	C	T	0.5538	-0.3076	0.051	1.653E-09	MCV	7,269	14,710	rs13203155	C	T	0.542658	0.01117	0.00183731	2.40E-07	exclude3
rs12718598	7:50428445	C	T	0.4939	0.3098	0.0523	3.15E-09	MCV	6,937	14,710	rs12718598	T	C	0.513091	-0.0031	0.00182115	0.069	
rs4535497	5:1107428	C	A	0.4346	-0.3103	0.0547	1.377E-08	MCV	6,171	14,710	rs4535497	C	A	0.428732	-0.0031	0.00187533	0.57	
rs34793991	10:46080590	C	A	0.0641	0.5839	0.1037	1.81E-08	MCV	1,755	14,710	rs34793991	C	A	0.930986	0.0131	0.00365247	7.00E-03	exclude3
rs12664956	6:135384188	C	T	0.37	0.2896	0.0526	3.557E-08	MCV	6,825	14,710	rs12664956	T	C	0.621803	-0.0033	0.00189778	0.088	
rs607203	6:139841653	C	T	0.96	-0.6945	0.1265	4.043E-08	MCV	1,167	14,710	rs607203	T	C	0.03756	0.00528	0.00485374	0.13	
rs35786788	6:135419042	G	A	0.739	-0.3501	0.0227	1.404E-53	MCH	5,567	14,715	rs35786788	T	C	0.740897	-0.0035	0.00209601	0.12	
rs855791	22:37462936	G	A	0.5667	0.2731	0.0205	1.558E-40	MCH	6,883	14,715	rs855791	A	G	0.438429	-0.006	0.00188032	0.00056	exclude3
rs1800562	6:26093141	G	A	0.9385	-0.4866	0.041	1.868E-32	MCH	1,685	14,715	rs1800562	G	A	0.92112	0.01162	0.00339748	0.0011	exclude3
rs2032451	6:26092170	G	T	0.8476	-0.2824	0.0276	1.419E-24	MCH	3,789	14,715	rs2032451	G	T	0.847802	-0.007	0.00254662	0.0035	exclude3
rs11964516	6:41860252	C	T	0.1774	0.1818	0.0266	8.268E-12	MCH	4,095	14,715	rs11964516	T	C	0.818167	0.00048	0.00236956	1	
rs7385804	7:100235970	C	A	0.3708	-0.1347	0.0214	3.047E-10	MCH	6,385	14,715	rs7385804	C	A	0.376323	0.00396	0.00187994	0.0054	
rs919797	19:4498157	G	A	0.4626	-0.1342	0.0221	1.183E-09	MCH	6,208	14,715	rs919797	G	A	0.436702	0.0013	0.00187632	0.62	
rs1410492	6:41907855	G	C	0.75	0.1377	0.0231	2.469E-09	MCH	5,370	14,715	rs1410492	G	C	0.738227	-0.0009	0.00208278	0.44	
rs140523	22:50962782	G	C	0.6126	0.1398	0.0236	3.261E-09	MCH	3,438	14,715	rs140523	C	G	0.390271	-0.0009	0.00190513	0.81	
rs13203155	6:109614844	C	T	0.5538	-0.1176	0.02	3.934E-09	MCH	7,272	14,715	rs13203155	C	T	0.542658	0.01117	0.00183731	2.40E-07	exclude3
rs218265	4:55408999	C	T	0.158	0.1677	0.0285	4.02E-09	MCH	3,515	14,715	rs218265	T	C	0.845215	-0.0049	0.0025781	0.28	
rs1799918	19:13002400	G	C	0.6365	-0.1251	0.0217	7.656E-09	MCH	6,180	14,715	rs1799918	G	C	0.621339	0.00308	0.0019108	0.12	
rs1800562	6:26093141	G	A	0.9385	-0.1247	0.0159	4.463E-15	MCHC	3,348	29,494	rs1800562	G	A	0.92112	0.01162	0.00339748	0.0011	exclude3
rs2032451	6:26092170	G	T	0.8476	-0.0798	0.0106	4.091E-14	MCHC	7,601	29,494	rs2032451	G	T	0.847802	-0.007	0.00254662	0.0035	
rs855791	22:37462936	G	A	0.5667	0.0578	0.0079	1.901E-13	MCHC	13,709	29,494	rs855791	A	G	0.438429	-0.006	0.00188032	0.00056	
rs35786788	6:135419042	G	A	0.739	0.0625	0.0048	3.344E-38	RBCC	5,565	14,712	rs35786788	T	C	0.740897	-0.0035	0.00209601	0.12	
rs218265	4:55408999	C	T	0.158	-0.0446	0.006	1.728E-13	RBCC	3,513	14,712	rs218265	T	C	0.845215	-0.0049	0.0025781	0.28	
rs7385804	7:100235970	C	A	0.3708	0.027	0.0045	3.004E-09	RBCC	6,383	14,712	rs7385804	C	A	0.376323	0.00396	0.00187994	0.0054	exclude4
rs1410492	6:41907855	G	C	0.75	-0.0284	0.0049	6.574E-09	RBCC	5,364	14,712	rs1410492	G	C	0.738227	-0.0009	0.00208278	0.44	
rs855791	22:37462936	G	A	0.5667	-0.1285	0.0214	1.897E-09	RDW	2,001	9,630	rs855791	A	G	0.438429	-0.006	0.00188032	0.00056	exclude3
rs35786788	6:135419042	G	A	0.739	0.1366	0.0236	7.153E-09	RDW	1,615	9,630	rs35786788	T	C	0.740897	-0.0035	0.00209601	0.12	

PAGE: Population Architecture using Genetic Epidemiology study

Trait	Acronym	Calculation	Units	Measures
Red blood cell count	RBCC	N/A	106 cells /mm3	Number of RBCs per unit of volume
Hemoglobin	HGB	N/A	g/dL	Blood level of hemoglobin
Hematocrit	HCT	N/A	%	Proportion of whole blood comprising RBCs
Mean corpuscular hemoglo	MCH	HGB*10 / RBC	picogram	Average hemoglobin protein per RBC (mass)
Mean corpuscular hemoglo	MCHC	HGB*100 / HCT	g/dL	Average hemoglobin protein per RBC (concentration)
Mean corpuscular volume	MCV	HCT*10 / RBC	femtoliter	Average size of RBC
Red cell distribution width (	RDW	SDMCV / MCV	%	Distribution of RBC size within an individual

exclude1：	palindromes;Using harmonise_data(exposure_dat, outcome_dat, action = 2) from TwoSampleMR.(default, conservative)
exclude2：	P-value in the outcome set is less than 5E-8,the SNP is considered to be directly related to the outcome.
exclude3:	Use MR-PRESSO to remove the outlier variants(with a P-value less than the MR-PRESSO outlier test threshold).
exclude4:	Continued removing other SNPs with P-values less than 1 from small to large in the MR-PRESSO outlier test and repeat MR analysis until there was no heterogeneity.

proxy SNP: take "rs9399137" as proxy SNP of "rs35786788".
take "rs9399137" as proxy SNP of "rs35786788".
effN: effective sample size

MR-PRESSO outlier

			RSSobs	Pvalue
HB	rs10253736	1	3.283721e-06	1
	rs17476364	2	1.903219e-05	1
	rs1800562	3	5.150201e-04	<0.008
	rs2032451	4	1.568676e-05	0.856
	rs35786788	5	3.137403e-06	1
	rs597808	6	2.761028e-05	0.392
	rs61523591	7	9.757520e-10	1
	rs855791	8	1.032579e-05	0.584

			RSSobs	Pvalue
MCH	rs11964516	1	1.655007e-07	1
	rs13203155	2	1.206874e-04	<0.012
	rs140523	3	2.771618e-06	1
	rs1410492	4	5.239097e-08	1
	rs1799918	5	6.632584e-06	1
	rs1800562	6	1.253913e-04	0.012
	rs2032451	7	8.720834e-05	0.024
	rs218265	8	3.535581e-05	0.18
	rs35786788	9	5.516160e-06	1
	rs7385804	10	1.198457e-05	0.684
	rs855791	11	7.861120e-05	<0.012
	rs919797	12	4.678652e-07	1

HCT	rs10253736	1	5.961858e-08	1
	rs17476364	2	7.833781e-07	1
	rs1800562	3	6.224565e-04	<0.009
	rs2032451	4	3.692128e-06	1
	rs2519093	5	6.887326e-04	<0.009
	rs35786788	6	2.843350e-06	1
	rs597808	7	1.054819e-05	1
	rs61523591	8	8.698216e-06	1
	rs855791	9	1.796933e-07	1

MCV	rs11964516	1	6.863049e-07	1
	rs12664956	2	1.009937e-05	1
	rs12718598	3	8.966404e-06	1
	rs13203155	4	1.393422e-04	<0.015
	rs140523	5	5.213815e-07	1
	rs1410492	6	1.494954e-06	1
	rs1800562	7	1.884419e-04	<0.015
	rs2032451	8	5.226639e-05	0.015
	rs218265	9	2.356022e-05	0.9
	rs34793991	10	1.737924e-04	<0.015
	rs35786788	11	2.713837e-05	0.255
	rs4535497	12	9.342021e-06	1
	rs607203	13	2.489703e-05	1
	rs7385804	14	1.885536e-05	0.255
	rs855791	15	4.169025e-05	<0.015

MR estimates of Hb on eBMD

Method	SNP(N)	BETA	SE	P-value	OR(95%CI)
Weighted median	7	0.064757351	0.013479868	1.56E-06	1.066(1.039-1.095)
Inverse variance weighted	7	0.065264903	0.010593005	7.22E-10	1.067(1.045-1.089)

MR estimates of HCT on eBMD

Method	SNP(N)	BETA	SE	P-value	OR(95%CI)
Weighted median	7	0.023111192	0.005395642	1.84E-05	1.023(1.012-1.034)
Inverse variance weighted	7	0.024105385	0.003974225	1.32E-09	1.024(1.016-1.032)

MR estimates of MCV on eBMD

Method	SNP(N)	BETA	SE	P-value	OR(95%CI)
Weighted median	8	0.005517073	0.002574431	0.032111	1.000(1.005-1.010)
Inverse variance weighted	8	0.004990208	0.002032235	0.014068	1.001(1.005-1.009)

MR estimates of MCH on eBMD

Method	SNP(N)	BETA	SE	P-value	OR(95%CI)
Weighted median	8	-0.00980864	0.005213095	0.059899	0.990(0.980-1.000)
Inverse variance weighted	8	-0.007494364	0.004996808	0.133658	0.992(0.982-1.002)

1a

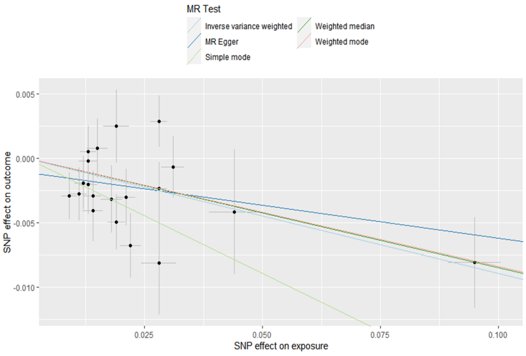
MR Test
Inverse variance weighted
MR Egger
Simple mode
Weighted median
Weighted mode
0.005
0.000
-0.005
-0.010
SNP effect on outcome
0.025
0.050
0.075
0.100
SNP effect on exposure

1b

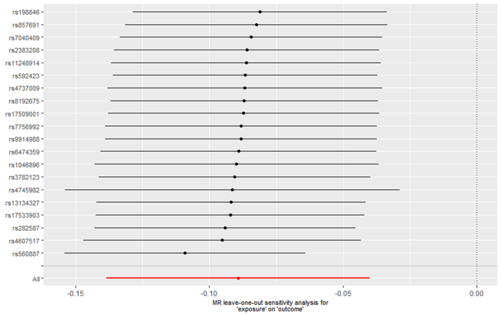
rs198846
rs857691
rs7040409
rs2383208
rs11248914
rs592423
rs4737009
rs8192675
rs17509001
rs7756992
rs9914988
rs6474359
rs1046896
rs3782123
rs4745982
rs13134327
rs17533903
rs282587
rs4607517
rs560887
All
-0.15
-0.10
-0.05
0.00
MR leave-one-out sensitivity analysis for
'exposure' on 'outcome'

1c

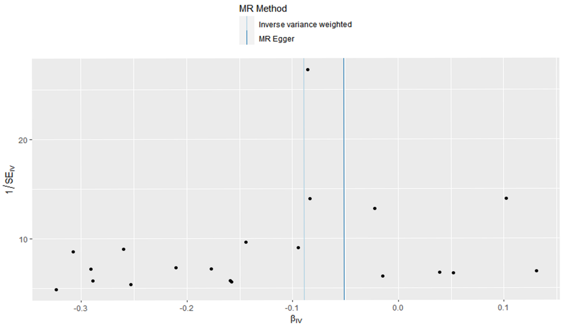
MR Method
Inverse variance weighted
MR Egger
20
10
$1/SE_{IV}$
-0.3
-0.2
-0.1
0.0
0.1
β_{IV}

2a

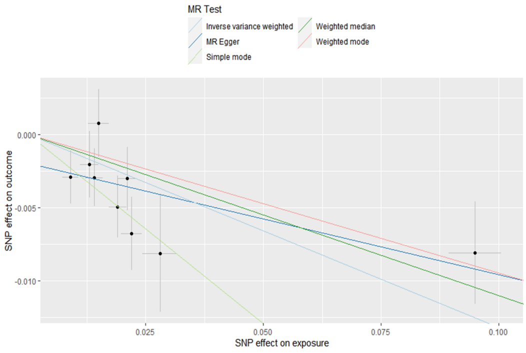
MR Test
Inverse variance weighted
MR Egger
Simple mode
Weighted median
Weighted mode
SNP effect on outcome
SNP effect on exposure
0.000
-0.005
-0.010
0.025
0.050
0.075
0.100

2b

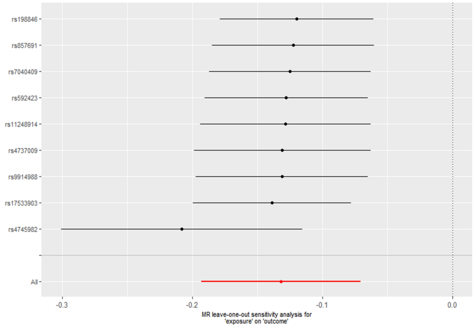
rs198846
rs857691
rs7040409
rs592423
rs11248914
rs4737009
rs9914988
rs17533903
rs4745982
All
-0.3
-0.2
-0.1
0.0
MR leave-one-out sensitivity analysis for
'exposure' on 'outcome'

2c

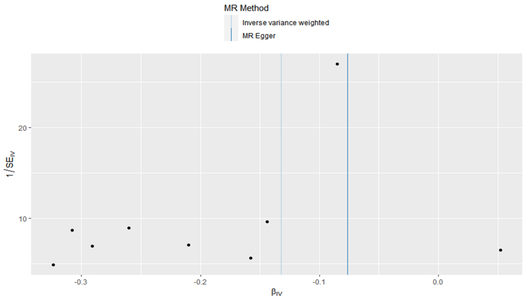
MR Method
Inverse variance weighted
MR Egger
1/SE_IV
β_IV
20
10
-0.3
-0.2
-0.1
0.0
