# Supplementary material for: HemoglobinA1c Is a Risk Factor for Changes of Bone Mineral Density: A Mendelian Randomization Study
Source: Front Endocrinol (Lausanne). 2022 Jul 18;13:942878. doi: 10.3389/fendo.2022.942878 (PMC9339617; doi:10.3389/fendo.2022.942878)
Supplement: Supplementary file 2 [file DataSheet_2.docx]

***Supplementary Material***

**Supplementary Data**

Following Guides by STROBE-MR: Guidelines for strengthening the reporting of Mendelian randomization studies

1. TITLE and ABSTRACT

Manuscript title

HemoglobinA1c is a risk factor for changes of bone mineral density: A Mendelian Randomization Study.

Abstract

Aims:

As a valuable blood glucose measurement, HemoglobinA1c (HbA1c) is of great clinical value for diabetes. However, in previous observational studies, studies on its effect on bone mineral density (BMD) have different results. Considering that observational studies often bring in some confounding, this study aimed to use Mendelian randomization (MR) to assess the effect of HbA1c on bone mineral density and fracture risk, and try to further explore whether this association was achieved through glycemic or non-glycemic factors.

Methods:

Take HbA1c measurement as exposure, and BMD estimated from quantitative heel ultrasounds (eBMD) and bone fractures as outcomes. Exposure statistics were taken from a large-scale transethnic meta-analysis genome-wide association study (GWAS) on HbA1C in individuals (the Meta-Analyses of Glucose and Insulin-Related Traits Consortium [MAGIC]) (n=159940). Outcome statistics were taken from GWAS based on UK Biobank data on estimated heel BMD (N=426284) and fracture (53184 cases and 373,611 non-cases). Two-Sample MR Analysis was conducted to assess the causal effect of HbA1C on heel BMD and risk fracture. Then, we performed the analysis using two subsets of these variants, one related to glycemic measurement (eg. fasting or 2-hour blood glucose) and the other to erythrocyte indices (eg. hemoglobin [Hb]).

Results:

Based on the inverse variance weighting (IVW) and weighted median methods of MR analysis, genetically increased HbA1C was associated with the lower heel eBMD (odds ratio [OR] 0.91 [95% CI 0.87, 0.96] per %-unit, P = 3 × 10−4(IVW); odds ratio [OR] 0.92 [95% CI 0.86, 0.97] per %-unit, P = 9 × 10−3(Weighted median)). Higher HbA1C was associated with lower heel eBMD when using only erythrocytic variants (OR 0.87 [0.82, 0.93], P=2× 10−5(IVW); OR 0.89 [0.83, 0.95], P=1 × 10−3(Weighted median)), However, when using only glycemic variants, this casual association does not hold. (OR 0.99 [0.88, 1.10], P=0.87 (IVW); OR 1.02 [0.90, 1.14], P=0.72(Weighted median)). In further MR analysis, we tested the association of erythrocytic traits with heel eBMD.

Conclusions:

Our study revealed that genetically increased HbA1C was associated with lower heel eBMD, and this causal link might achieve through non-glycemic pathways (erythrocytic indices).

INTRODUCTION

2. Background

The effect of HbA1c on bone mineral density and fracture risk has been inconclusive, the different or even opposite conclusions in observational studies are thought-provoking. Some studies suggest that high levels of HbA1c lead to decreased bone mineral density [7,8,9], and lead to higher fracture risk [10]. But other studies have found that patients with higher HbA1c level usually had higher BMD [11].

These conflicting conclusions just show that observational studies are often subject to confounding and bias caused by various factors.

Mendelian randomization analysis can offer substantial evidence for the influence of modifiable risk variables on illness or poor health, and overcome some of the limitations of classic observational studies. It selected publicly available results from large genome-wide association studies (GWAS), using germline genetic variation as an instrumental variable (IV) for exposure, to investigate causal relationships between risk factor "exposure" and disease "outcome" [12].

In our study, we selected HbA1c as an instrumental variable (IV) to study its causal effect on eBMD and fracture risk. Since HbA1c levels are not only determined by glycemia, nonglycemic determinants of HbA1C that are intrinsic to erythrocyte [13,14] may also works, we further explored whether this relationship is driven by glycemic or erythrocytic factors.

3. Objectives

The purpose of this study was to explore the causal effect of HbA1c on bone mineral density and fracture risk from a genetic perspective with two-sample Mendelian randomization analysis. HbA1c is a clinically important and commonly used diabetes indicator. Through this study, genetic evidence for osteoporosis prevention guidelines based on HbA1c levels can be enhanced; HbA1c could also be a potential reference marker for osteoporosis complications in patients.

And this study is the first report of the genetic association about HbA1c on bone mineral density and fracture risk.

METHODS

4. Study design and data sources

HbA1c, measured in mmol/mol using HPLC (Bio-Rad Variant II Turbo analysers, Bio-RadLaboratories, USA) and generally reported as a NGSP (National Glycohemoglobin Standardization Program) percent [15]. Regarding eBMD and fracture risks, they were determined by bone mineral density estimated from quantitative heel ultrasounds and from bone fractures respectively. The fracture cases were characterized using the 10th version of the International Classification of Diseases codes. Malignant pathological fractures, atypical femoral fractures, periprosthetic fractures and healed fractures were exclude. And the fracture data were questionnaire-based self-reported fractures and from Hospital Episodes Statistics [16].

In this study, we selected HbA1c as an instrumental variable to investigate its causal effect on eBMD and fracture risk. Since HbA1c variants can be classified as “glycemic” or “erythrocytic” [17].

The data source of HbA1c was obtained from MAGIC (the Meta-Analyses of Glucose and Insulin-Related Traits Consortium), a genome-wide association study (GWAS) of 123665 participants of European ancestry (no overlap with the UK Biobank), and 159940 participants of mixed ancestries without diabetes were included in the original GWAS study. With a similar ratio of males to females, the average age of the participants in this GWAS was about 50 years, it also took into account age and gender, as well as study-specific covariates and genetic controls [17].

The data sources of eBMD and fracture were downloaded from the Genetic Factors for Osteoporosis Consortium website (GEFOS, http://www.gefos.org/). And we choose the largest GWAS of BMD to date that consisted of 426824 white British UK Biobank participants. Regarding to eBMD, with 233185 females and 193639 males, the average age of the white British participants in this GWAS was about 57 years; About fracture, its study data included 53184 positive cases and 373611 controls of white British participants, the average age was about 58.2 years (39-73 years old). [16]

5. Assumptions

Three core IV assumptions for this Mendelian randomization analysis (relevance, independence and exclusion restriction):

1)The genetic instruments should be associated with the exposure, here HbA1c.

2)The genetic instruments should not be confounded by factors affecting the exposure-outcome relationship.

3)The genetic instruments should only affect the outcome (eBMD) via affecting the exposure (HbA1c).

6. Statistical methods: main analysis

SNP selection and validation

The genetic instrumental variables (IVs) of HbA1c were selected from the genome-wide association study (GWAS) of 123,665 participants of European ancestry (no overlap with the UK Biobank) [17]. Here, we process instrumental variables as follows: At first, IVs associated with HbA1c should reach genome-wide significance (P < 5 ×10−8). Secondly, since IVs in strong LD (linkage disequilibrium) may result in skewed findings, it is important to make certain that none of the exposure's IVs are in LD. The clumping process (R2 < 0.001, window size = 10,000 kb) was performed among the 1000G European reference panel to exclude the IVs with strong LD. Thirdly, these above-selected IVs were extracted from the eBMD and fracture associated GWAS summary statistics. If a particular requested SNP (associated with HbA1c, set as target SNP) is not presented in the outcome GWAS summary statistics, then a SNP (as a proxy) that is in LD with the target SNP will be searched for instead with LD r2 > 0.8. Then the proxy SNP was returned on the outcome GWAS summary data, along with its effect, the effect allele, and the corresponding allele for the target SNP. Fourthly, the harmonization of the effect was performed to make certain that the same SNP effect of exposure and outcome data correspond to same allele. (harmonise_data(exposure_dat, outcome_dat, action = 2)) The detailed screening steps are in Supplementary Table1. After these rigorous selections, these SNPs were used for subsequent analysis.

The selected instrumental SNPs should substantially connect with exposure, we assessed the F statistic to see if there was a weak instrumental variable bias, meaning that the genetic variations chosen as instrumental factors had a weak correlation with exposure. All IVs’ F statistic is greater than 30, which showed in Supplementary Table2, so the possibility of weak instrumental variable bias is small [18].

MR estimation

Using the above data, we ran two-sample MR analysis to generate overall estimates of the effect of HbA1c on eBMD. The IVW method combines Wald estimates for each SNP to provide overall estimates of the effect of HbA1c on eBMD using a meta-analysis approach (i.e, the β coefficient of the SNP for eBMD divides by the β coefficient of the SNP for HbA1c) [19]. If the IV2 assumption is not violated (no horizontal pleiotropy), or the horizontal pleiotropy is balanced, IVW linear regression can provide an unbiased causal estimate [20].The MR-PRESSO approach detects and corrects outliers in IVW linear regression, and the MR-PRESSO outlier test requires at least half of the variations to be valid, has balanced pleiotropy, and is based on the Instrument Strength Independent of Direct Effect (InSIDE) condition, which states that instrument-exposure and pleiotropic effects are uncorrelated [21]. Based on the premise of InSIDE, the MR-Egger regression conducts a weighted linear regression of the result coefficients on the exposure coefficients [22]. Even if all of the genetic variants are invalid IVs, the InSIDE assumption yields a valid test of the null causal hypothesis and a consistent causal effect estimate [22]. Nevertheless, MR-Egger estimations are subject to error and are heavily influenced by outlying genetic variants. The Weighted Median approach, which does not require the InSIDE assumption, has been shown to outperform the MR-Egger estimate because of its better causal effect detection power and lesser type I error [23]. Therefore, the main results are based on the IVW and WM methods.

7. Assessment of assumptions

Firstly, all the above-selected SNPs were performed with MR analysis, If the MR-PRESSO analysis reveals a significant horizontal pleiotropy, the outlier variants will be removed (with a P-value less than the MR-PRESSO outlier test threshold). After this step, if the heterogeneity was still significant, we continued removing other SNPs with P values less than 1 from small to large in the MR-PRESSO outlier test and repeat MR analysis until there was no heterogeneity. Furthermore, in order to identify potentially relevant SNPs, we use the "leave-one-out" sensitivity analysis in which the MR was repeated but each SNP was removed one at a time.

8. Sensitivity analyses

The MR-Egger method can determine if genetic variants have pleiotropic effects on outcomes that differ from zero on average [24], so it was used to examine the potential pleiotropic effects of the SNPs we selected. The MR‐PRESSO recognizes the existence of variant effect sizes that are outliers then removing them to correct pleiotropy [25], and it seeks to reduce heterogeneity in the assessment of the causal effect by eliminating SNPs that contribute disproportionately more than expected. In the MR-PRESSO study, the number of distributions was set to 1000. To determine heterogeneity, we employed the IVW and MR-Egger regression. The heterogeneities were measured using the Cochran Q statistic, with a P value of 0.05 indicating significant heterogeneity. Furthermore, we performed a “leave-one-out” sensitivity analysis to identify potentially influential SNPs.

9. Software and pre-registration

Based on the TwoSampleMR Guideline and the MR-PRESSO vignette, all statistical analyses were conducted with TwoSampleMR package (v0.5.6) and MRPRESSO package (v1.0) performed in R version 4.0.3 statistical software (using RStudio v1.3.1093). The study protocol and details were not pre-registered.

RESULTS

10. Descriptive data

HbA1c associated SNPs with a GWAS significance (P < 5×10-8) and LD clumped were extracted. There were 20 SNPs of HbA1c associated with eBMD, 9 SNPs of erythrocytic variants of HbA1c associated eBMD, and 34 SNPs of HbA1c associated with bone fracture. Corresponding information of IVs in the outcome dataset was matched.

The exposure data(MAGIC) has no sample overlap with outcome data(UKBB).

11. Main results

Based on the inverse variance weighting (IVW) and Weighted median methods of MR analysis, genetically increased HbA1C was associated with the lower heel eBMD (odds ratio [OR] 0.91 [95% CI 0.87, 0.96], P = 3 × 10−4(IVW); odds ratio [OR] 0.92 [95% CI 0.86, 0.97], P = 9 × 10−3(Weighted median)), the results of the forest plot are shown in Figure1.

Higher HbA1C was associated with lower heel eBMD when using only erythrocytic variants (OR 0.87 [0.82, 0.93], P=2× 10−5(IVW); OR 0.89 [0.83, 0.95], P=1 × 10−3(Weighted median)). However, when using only glycemic variants, this casual association does not hold (OR 0.99 [0.88, 1.10], P=0.87 (IVW); OR 1.02 [0.90, 1.14], P=0.72(Weighted median)). The results of the forest plot are shown in Figure1.

12. Assessment of assumptions

The results indicated that HbA1c impacted the heel bone mineral density. The MR‐PRESSO recognizes the existence of variant effect sizes that are outliers then removing them to correct pleiotropy. Heterogeneity results from the test demonstrated that there was no heterogeneity in the analysis.

13. Sensitivity and additional analyses

In the MR-PRESSO study, the number of distributions was set to 1000. To determine heterogeneity, we employed the IVW and MR-Egger regression. The heterogeneities were measured using the Cochran Q statistic, with a P value of 0.05 indicating significant heterogeneity. Furthermore, we performed a “leave-one-out” sensitivity analysis to identify potentially influential SNPs.

To further clarify the causal effect of the non-glycemic fraction of HbA1c on eBMD, we did additional MR analysis to explore the direct causal link of hemoglobin on eBMD.

DISCUSSION

14. Key results

In the present study, GWASs summary statistics were used to estimate the causal effect of HbA1c on eBMD through standard MR analysis. Sensitivity analyses were also conducted to assess the reliability and stability of the results.

Based on the inverse variance weighting (IVW) and weighted median methods of MR analysis, genetically increased HbA1C was associated with the lower heel eBMD (odds ratio [OR] 0.91 [95% CI 0.87, 0.96] per %-unit, P = 3 × 10−4(IVW); odds ratio [OR] 0.92 [95% CI 0.86, 0.97] per %-unit, P = 9 × 10−3(Weighted median)). Higher HbA1C was associated with lower heel eBMD when using only erythrocytic variants (OR 0.87 [0.82, 0.93], P=2× 10−5(IVW); OR 0.89 [0.83, 0.95], P=1 × 10−3(Weighted median)), However, when using only glycemic variants, this casual association does not hold. (OR 0.99 [0.88, 1.10], P=0.87 (IVW); OR 1.02 [0.90, 1.14], P=0.72(Weighted median)). In further MR analysis, we tested the association of erythrocytic traits with heel eBMD.

15. Limitations

There are several limitations to this research. Among the MR statistical methods, although the causal effect of exposure on outcome was consistent in both IVW and WM test methods, the results of MR-Egger were less convincing. The instrumental variables we selected have undergone very strict procedures and qualified sensitivity analysis, and horizontal pleiotropy was detected and corrected by MR-PRESSO; each instrumental variable was determined to be not a weak instrumental variable by F test (all greater than 30, it is generally considered that greater than 10 is not a weak instrumental variable), and it was reported that the estimated efficiency of MR-Egger is lower than WME and IVW [43], so in conclusion, for this present study, the results of the IVW method are more reliable and credible. Secondly, our findings were limited to the heel bone mineral density. When we used outcome sets of BMD at other body sites (LS-BMD; FN-BMD; FA-BMD), the relationship between HbA1c and BMD was not statistically significant, so it should be cautious to extrapolate the causal relationship of HbA1c on the heel BMD to whole-body BMD, and we speculate that there may be site differences in the effect of HbA1c on BMD, the reasons behind need to be further explored. Thirdly, our findings pertain to impacts of HbA1c in the non-diabetic range, there is no way to draw conclusions concerning genetic impact of HbA1c in diabetics, and we recommend exercising caution when projecting our findings to the HbA1c distribution's extremes. So, using it as a genetic hypothesis reference for clinical diabetes research is more rigorous and scientific. Fourthly, when studying the causal effect of genetically altered Hb levels and other erythrocytic traits on eBMD, we cannot extrapolate results to specific blood diseases or a specific type of anemia, which requires further exploration. Finally, this study is confined to the European population; whether this association exists in other groups warrants additional investigation.

16. Interpretation

This finding has many intriguing implications:

First of all, the results of this study can explain the conflicting views in previous observational studies to a certain extent. In epidemiological studies, when investigating the effect of HbA1c on bone mineral density, many confounding factors often need to be corrected, such as gender, age, BMI, etc. After these corrections, some studies have found that HbA1c can lead to a decrease in bone density, while other studies showed that HbA1c results in an increase in bone density or there is no correlation between them. However, none of these studies adjusted for Hb levels for confounders, for our results suggest that HbA1c acts as a risk factor for BMD loss, primarily through erythrocytic pathways. Therefore, it is necessary to consider the influence of Hb and some other erythrocytic characteristics when conducting research on HbA1c.

Secondly, HbA1c, regarded as the gold standard for predicting related risks for complications of diabetes mellitus, has demonstrated unassailable value in microvascular and macrovasculara disorders [33]. In adolescents with T1D, higher HbA1c variability predicts retinopathy, early nephropathy, and cardiac autonomic neuropathy [34]. Controlling HbA1c levels appears to prevent proliferative retinopathy and persistent macroalbuminuria for up to 20 years [35]. In T2D individuals, increased HbA1c is linked to diabetic peripheral neuropathy and might be used as a reliable predictor of diabetic peripheral neuropathy in these patients [36]. Despite its limitations, HbA1c remains the preferred glycemic biomarker in diabetic patients with advanced chronic kidney disease. An appropriate range of HbA1c can help develop a glycemic control program and reduce the risk of mortality and hypoglycemia to a certain extent [37]. Similarly, osteoporosis-related complications of diabetes have also attracted more and more attention in recent years. Our study provides genetic evidence and support for HbA1c as its potential predictor and control indicator, while the specific control range and level still need further clinical studies.

Moreover, our study demonstrates that genetically elevated Hb levels are associated with higher heel eBMD, similar studies have previously reported. In a cross-sectional investigation found a link between low BMD and low Hb levels, as well as the occurrence of anemia in postmenopausal women [38]. Another cross-sectional investigation showed that Hb levels and BMD were found to have a positive association in adult men [39]. Correction of low Hb levels may have a preventive function in the prevention of osteoporosis [40]. However, if the population is located in diabetic patients, there are few reports on the effect of Hb decrease on osteoporosis. Diabetics, on the other hand, are more likely to develop anemia and osteoporosis [41]. As a result, research into the link between anemia and osteoporosis in diabetics is required. A retrospective cross-sectional study showed that men and women with the lowest hemoglobin levels had a greater percentage of osteoporosis. And BMD was also correlated with hemoglobin levels in both sexes. They alert that diabetic with anemia (men with hemoglobin below 120 g/L and women with hemoglobin below 110 g/L) should also be wary of osteoporosis [42].

In some ways, the findings of our research support this hypothesis. Our study suggests that hereditary elevated HbA1c can lead to a decrease in heel bone mineral density, and this effect occurs mainly through erythrocyte pathways such as Hb decrease, so we should pay attention to and prevent osteoporosis complications in patients with higher HbA1c levels, especially when combined with some blood diseases (such as anemia and other diseases that may lead to a decrease in hemoglobin).

17. Generalizability

Releasing the potential causal effect between HbA1c and eBMD could promote osteoporosis preventive guidelines, give precise prescriptions for the target population, and eventually reduce fracture risk of those individuals who with high HbA1c level.

Our findings were limited to the heel bone mineral density. When we used outcome sets of BMD at other body sites (LS-BMD; FN-BMD; FA-BMD), the relationship between HbA1c and BMD was not statistically significant, so it should be cautious to extrapolate the causal relationship of HbA1c on the heel BMD to whole-body BMD.

Our findings pertain to impacts of HbA1c in the non-diabetic range, there is no way to draw conclusions concerning genetic impact of HbA1c in diabetics, and we recommend exercising caution when projecting our findings to the HbA1c distribution's extremes.

This study is confined to the European population; whether this association exists in other groups warrants additional investigation.

OTHER INFORMATION

18. Funding

This study is supported by research grants from Zhejiang Natural Science Foundation (No. LQ21H060006), Zhejiang Province Medical and Health project (NO.2020391395), the National Natural Science Foundation of China (No.82001461), and the fellowship of China Postdoctoral Science Foundation (No.2020M671758).

19. Data and data sharing

The article/Supplementary Material contains the original contributions presented in the study; further questions should be directed to the corresponding author(s).

20. Conflicts of Interest

Declaration of interest: none.
